# Familiarity, but not Recollection, Supports the Between-Subject Production Effect in Recognition Memory

**DOI:** 10.1037/cep0000089

**Published:** 2016-06

**Authors:** Jonathan M. Fawcett, Jason D. Ozubko

**Affiliations:** 1 MRC Cognition and Brain Sciences Unit, Cambridge, United Kingdom; 2 Rotman Research Institute, Baycrest, Ontario

**Keywords:** production effect, memory, distinctiveness, recollection, familiarity, effet de la production, mémoire, disinctivité, souvenir, familiarité

## Abstract

Five experiments explored the basis of the between-subjects production effect in recognition memory as represented by differences in the recollection and familiarity of produced (read aloud) and nonproduced (read silently) words. Using remember-know judgments (Experiment 1b) and a dual-process signal-detection approach applied to confidence ratings (Experiments 2b and 3), we observed that production influences familiarity but not recollection when manipulated between-subjects. This is in contrast to within-subject designs, which reveal a clear effect of production on both recollection and familiarity (Experiments 1a and 2a). Our findings resolve contention concerning apparent design effects: Whereas the within-subject production effect is subserved by separable recollective- and familiarity-based components, the between-subjects production effect is subserved by the familiarity-based component alone. Our findings support a role for the relative distinctiveness of production as a means of guiding recognition judgments (at least when manipulated within-subjects), but we also propose that production influences the strength of produced items, explaining the persistence of the effect in between-subjects designs.

## Abstract (fr)

Cinq expériences visaient à explorer les fondements de l’effet de la production inter-sujets sur la mémoire de reconnaissance, tels que représentés par les différences entre le rappel et la familiarité des mots produits (lus à haute voix) et non produits (lus en silence). Au moyen de jugements souvenir-savoir (Expérience 1b) et une démarche en deux temps de détection du signal appliquée aux cotes de certitudes (Expériences 2b et 3), nous avons observé que la production influait sur la familiarité, mais non sur le rappel lors d’une manipulation inter-sujets. Ce résultat est contraire aux cadres intra-sujets, où se révèle clairement l’effet de la production à la fois sur le rappel et la familiarité (Expériences 1a et 2a). Nos résultats permettent de résoudre les différends au sujet des effets apparents des cadres : si l’effet de la production intra-sujets est favorisé par des éléments basés sur le rappel et la familiarité dissociables, l’effet de production inter-sujets est favorisé uniquement par l’élément basé sur la familiarité. Nos résultats soutiennent l’apport de la distinctivité relative de la production comme moyen de guider les jugements de reconnaissance (tout au moins par suite d’une manipulation chez les intra-sujets). Nous proposons aussi que la production influe sur la force des éléments produits, ce qui explique la persistance de l’effet dans les cadres inter-sujets.

When he reached the office, about nine o’clock in the morning, the first thing he did was to pick up a newspaper, spread himself out on an old sofa, one leg on a chair, and read aloud, much to my discomfort. Singularly enough Lincoln never read any other way but aloud. This habit used to annoy me almost beyond the point of endurance. I once asked him why he did so. This was his explanation: “When I read aloud two senses catch the idea: first, I see what I read; second, I hear it, and therefore I can remember it better” (William H. Herndon & Jesse W. Weik, 1896, *Abraham Lincoln: The True Story of a Great Life, Volume 2, accessed via http://www.gutenberg.org/files/38484/38484-h/38484-h.htm*).

It has long been known that the act of reading words aloud improves memory for those words relative to other words that have been read silently (e.g., Conway & Gathercole, 1987; Hopkins & Edwards, 1972). This finding was initially used to explore the influence of frequency on memory (Ekstrand, Wallace, & Underwood, 1966; Hopkins, Boylan, & Lincoln, 1972); however, interest has been revived recently owing to the thorough review and rebranding of the effect by MacLeod, Gopie, Hourihan, Neary, and Ozubko (2010). They referred to the finding as the *production effect*, and since that time it has been determined that many forms of production (e.g., saying, writing, singing; e.g., Forrin, MacLeod, & Ozubko, 2012; Quinlan & Taylor, 2013) improve subsequent retention of produced items relative to nonproduced items (typically, read silently).

While the mechanisms underlying the production effect remain under debate (e.g., Bodner & Taikh, 2012; Fawcett, 2013), much of the research suggests that distinctiveness (or a *distinctiveness heuristic*) plays a key role. According to this view, participants retain some element of the production episode for each produced item and these “production traces” may then be retrieved or reconstituted at test to differentiate produced study items from nonproduced foil items and (potentially) silent items (e.g., MacLeod et al., 2010): If the participant remembers having recently produced a given test item it is likely that they studied it, because it is unlikely that they would have recently produced a foil item (the opposite inference may also be made; Dodson & Schacter, 2001). This theoretical perspective connects with broader retrieval-based theories of memory for “distinct” items, which state for example that the features of distinct items may benefit memory performance due to “. . . enhanced discriminability of the target from possible candidates generated at recall or provided at recognition . . . [or through] the use of these unusual features to guide access or direct retrieval” (McDaniel & Geraci, 2006, p. 67). Supporting this prediction, the production effect in a list-discrimination task disappears when participants are instructed to produce the foil items prior to the study phase (Ozubko & MacLeod, 2010; cf. Bodner & Taikh, 2012).

However, support for a retrieval-based distinctiveness account has not been universal. From the beginning, the production effect was thought to occur only when manipulated within-subjects as opposed to between-subjects (e.g., Dodson & Schacter, 2001; Hopkins & Edwards, 1972; MacLeod et al., 2010). Within the modern literature, this finding was quickly interpreted as support for the distinctiveness account, with the reasoning that participants are unlikely to use production as a retrieval strategy unless juxtaposed against items that were not themselves produced (e.g., MacLeod et al., 2010; Ozubko & MacLeod, 2010). However, a series of recent meta-analyses have revealed a surprisingly consistent between-subjects production effect when these studies are aggregated (Bodner, Taikh & Fawcett, 2014; Fawcett, 2013). This finding has since been replicated, although results have been mixed with respect to whether the magnitude of the production effect is comparable across design—or whether it is larger for within-subject than for between-subjects designs (e.g., Bodner, Jamieson, Cormack, McDonald, & Bernstein, in press; Bodner et al., 2014; Fawcett, 2013; Forrin, Groot, & MacLeod, 2016). While the existence of a between-subjects production effect does not itself undermine a distinctiveness-based account, it eliminates one positive argument that was often cited in its defence. Bodner and Taikh (2012) cast further doubt on this framework by revealing biases in the list-discrimination task used to demonstrate that producing foil items can eliminate the production effect (Ozubko & MacLeod, 2010). Finally, Bodner, Taikh and Fawcett (2014; see also, Forrin & MacLeod, in press; Jones & Pyc, 2014; Lambert, Bodner, & Taikh, in press) demonstrated that the within-subject production effect may be characterised at least in part by a *decrease* in performance for nonproduced items relative to a pure-list nonproduced baseline condition, as originally noted by Hopkins and Edwards (1972).

The subjective experiences reported by participants at test suggest that a distinctiveness-based strategy is unlikely to provide an exhaustive explanation of the production effect. Dual-process accounts propose that memory arises from a combination of two separable processes (Mandler, 1980; Tulving, 1985; for a review, see Yonelinas, 2002): Familiarity is often characterised as an undifferentiated feeling that a stimulus was recently experienced, and arises when a stimulus is processed fluently. In contrast, recollection is typically construed as the ability to vividly and consciously reexperience a past event and the context surrounding it. The standard distinctiveness account proposes that production arises from the strategic use of access to the production trace at test, and could imply that the production effect is driven by recollection. To test this possibility, Ozubko, Gopie, and MacLeod (2012) used either remember-know judgments (e.g., Tulving, 1985) or confidence ratings (e.g., Yonelinas, 1994) to measure separately the influence of production on recollection and familiarity. For both test procedures, production improved recollection and familiarity to a similar degree: Ozubko et al. (2012) interpreted the effect of production on recollection as arising from the strategic use of distinctive information at test (consistent with the distinctiveness account); however, they speculated that the effect of production on familiarity instead arises from attentional processes at encoding (see also Fawcett, 2013).

The idea that the production effect could arise from a combination of relative distinctiveness alongside some other mechanism provides a possible explanation for the reported nonreplications of the production effect in between-subjects designs. If relative distinctiveness were a recollective phenomenon observable only when production was manipulated within-subjects, but production also improved familiarity irrespective of study design, the production effect would then be expected to be larger (and therefore more reliable) when manipulated within-subjects. Importantly, whereas this account, which we will call the *dual-process account,* predicts that the within-subjects production effect should emerge for measures of both familiarity and recollection, the between-subjects production effect should emerge for measures of familiarity but not recollection. By supporting this framework, we will provide a more coherent explanation of why the between-subjects production effect arises, and why it is less reliable than the within-subject production effect in recognition memory.

Our goal was thus to investigate whether the production effect differentially relies on recollection and familiarity in within-subject and between-subjects designs. Experiments 1a and 1b compared the magnitude of the production effect, as indexed by estimates of recollection and familiarity across between- and within-subject designs using remember-know responses (Gardiner, 1988; Tulving, 1985). Experiments 2a and 2b next replicated our results using estimates of recollection and familiarity derived from simple confidence ratings using a dual-process signal detection framework (Yonelinas, 1994, 1997). Experiment 3 provided a final replication of our between-subjects design in a large, online sample. We then conducted a meta-analysis to compare formally the magnitude of the production effect captured by each dependent measure as a function of study design. To foreshadow our results, we observed a reliable production effect for both recollection and familiarity across each of our within-subject experiments (Experiments 1a and 2a) but a production effect only for familiarity for each of our between-subjects experiments (e.g., Experiment 3).

## Experiment 1a: Within-Subject Design With Remember-Know Judgments

The purpose of Experiment 1a was to provide a baseline for further experiments by replicating the findings of Ozubko et al. (2012, Experiment 1), which used remember-know judgments to demonstrate an effect of production on both recollection and familiarity in a within-subject design. Therefore, the current experiment manipulated production within-subjects and probed memory using remember-know judgments.

### Method

#### Participants

A sample of 25 participants enrolled at Dalhousie University took part in exchange for partial course credit.

#### Stimuli and apparatus

All experimental procedures were presented using custom software developed in the Python programming language (www.python.org) with the Pygame development library (www.pygame.org) loaded on a 24-inch iMac computer running Mac OSX Snow Leopard, version 10.6. Responses were recorded via a standard Macintosh Universal Serial Bus keyboard. Words and fixation stimuli were presented at centre in Arial size 42-point font against a black background.

Stimuli consisted of 240 words sampled at random from the MRC Psycholinguistic Database (Wilson, 1988; see Supplementary Online Materials). Words ranged from three to 12 letters in length (*M* = 6.08, *SD* = 1.89) with Kučera-Francis word frequencies from one to 231 (M = 69.48, *SD* = 127.87; Kučera-Francis, 1967).^1^ For each participant the stimuli were randomly distributed across the silent, aloud, and foil conditions resulting in two lists containing 60 words and one list containing 120 words. During the study phase, words were presented in either purple (RGB: 128, 0, 128) or green (RGB: 0, 100, 0) to denote which items participants were to read silently or aloud. For half of the participants, purple instructed them to read the item silently and green instructed them to read the item aloud; these instructions were reversed for the remaining participants. During the test phase, study and foil words were presented in white (RGB: 255, 255, 255).

#### Procedure

##### Study phase

During the study phase, the 120 items were presented one at a time in a randomized order. Each study phase trial consisted of a fixation stimulus (“+”) lasting 500 ms, followed by the study item for 2,000 ms.

##### Test phase

Following the study phase, participants were tested for their memory of the study items using the remember-know procedure (Tulving, 1985) as described by Ozubko et al. (2012, Experiment 1). Briefly, the remember-know procedure involves participants identifying studied items as either “remembered” or “known” to indicate recollection or familiarity, respectively. In this experiment, it was explained to participants that when they recognised an item, that memory could be supported by either recollection or familiarity. When items are supported by recollection, participants should be able to “see” the item in their minds eye, and remember what it was like when they first encountered it (e.g., what they were thinking about or felt when they saw the item, what items had come before or after it). In these cases, participants were instructed to provide a remember response. In other cases, participants would be able to recognise a word as one they had studied, but they would not have access to any of these subjective details. In these cases participants were instructed to provide a know response. Importantly, our instructions emphasised that know responses did not simply encompass low confidence responses and that it indeed was possible to be highly certain an item was studied but simply not have the subjective details of what happened during the specific study episode where the item was seen. Hence, remember-know judgments were to be made on the basis of what participants could remember about an item, rather than confidence.

Participants were also informed that at the end of the experiment they would be asked to explain what kinds of details came to mind for items they identified as remembered. Strict remember-know instructions such as these have been shown to produce remember and know responses that converge with estimates of recollection and familiarity drawn from other sources such as from dual-process signal detection analyses of receiver-operating characteristic (ROC) curves (see Rotello, Macmillan, Reeder, & Wong, 2005; Yonelinas, Dobbins, Szymanski, Dhaliwal, & King, 1996; Yonelinas, 2001). Participants were informed that they would be presented with each of the words from the study phase (regardless of production condition) as well as an equal number of “new” words that they had not studied (i.e., foils). Test items were presented one at a time in a randomized order, preceded by a 500 ms fixation stimulus (“+”). For each of the 240 test items, participants registered a “remember,” “know,” or “new” response using the “a,” “s,” or “d” keys, respectively. Responses were self-paced and participants were instructed to respond to each test item as accurately as possible. Following completion of the test phase, participants further completed a strategy questionnaire, which is analysed and reported in the Supplementary Online Materials.

#### Statistical tools

Two features of the present experiments motivated us to adopt a fully Bayesian approach in handling our results (for further discussion, see Dienes, 2011; Fawcett, Lawrence, & Taylor, 2016). The first concerns the binary response measures (such as recognition accuracy) used in our initial experiments. Although such data are commonly aggregated into proportions prior to analysis (e.g., using an Analysis of Variance; ANOVA), simulations have consistently demonstrated the superiority of statistical models that treat the raw binary scores as arising from a binomial distribution (e.g., Dixon, 2008; Jaeger, 2008). These models are efficiently implemented within a Bayesian framework. For this reason we have analysed all binary measures using multilevel logistic regression within the *Stan* modelling language (Stan Development Team, 2013).

The second feature motivating our use of Bayesian statistics is that a major component of our theoretical framework rests upon our ability to draw conclusions in the context of a *null* effect with regards to production manipulations and estimates of recollection in between-subjects designs. Whereas frequentist statistics of the sort generally used within the social sciences are incapable of drawing conclusions from a nonsignificant statistical test, this is not an issue for Bayesian statistics—which permit interpretation of subjective evidence on a continuous scale (see Kruschke, 2010). In the absence of a mechanism capable of interpreting the presence of a *null* effect within the frequentist tradition, we have therefore opted to use a fully Bayesian framework. We have particularly embraced the parameter estimation approach wherein emphasis is placed upon estimating the credible range of the parameters corresponding to our hypotheses (for introductions, see Gelman & Hill, 2007; Kruschke, 2010).

For the interested reader, we have provided further description of our statistical approach in the Supplementary Online Materials (see also Fawcett et al., 2016). However, for practical purposes our results may be interpreted as any other regression model. For each, we provide the intercept and relevant slopes for the reported model in-text. However, the critical statistical contrasts (e.g., comparing the aloud and silent conditions) may be interpreted graphically: In such cases, the median difference is surrounded by the highest-density interval (HDI; Kruschke, 2010) calculated for the posterior distribution of the relevant parameter. The HDIs represent the most credible values of the estimated parameter given the combination of prior beliefs for those parameters and the current data. If an HDI corresponding to a comparison or parameter fails to include a particular value (e.g., 0), then this value is interpreted as being not credible given the model. Additional probabilistic statements can also be derived as necessary; for example, if 75% of the credible values fall above 0, we can state that we are 75% confident that the true value of the parameter in question is positive. While we have tried to make our models easy to follow, we recognise that some readers might wish to see our analyses framed in a more familiar light. For this reason, frequentist models (i.e., ANOVAs) are provided in the Supplementary Online Materials alongside the raw condition means. However, it is our opinion that the analyses provided in-text are preferable.

### Results and Discussion

#### Old responses

As an initial analysis, the remember and know responses were collapsed into old responses (representing having made either response), so that hits and false alarms could be calculated. We then applied a multilevel logistic regression model with item type (foil, silent, aloud) as a fixed effect. Because item type was a categorical variable, the silent and aloud conditions were each dummy coded as 0 or 1 with foil serving as the relevant intercept. As such our model estimated three fixed-effect coefficients—the intercept (i.e., the logit transformed proportion of false alarms to foil items) as well as contrasts between this intercept and each of the silent and aloud conditions (i.e., their respective slope coefficients).

**Figure 1 fig1:**
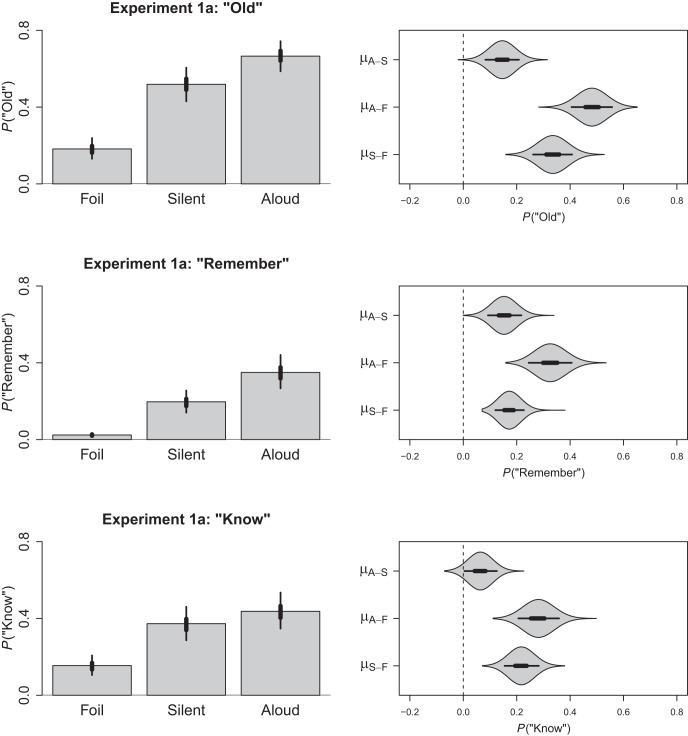
The left column depicts the back-transformed estimated proportion of old, remember, and know responses for Experiment 1a as a function item type (foil, silent, aloud). The right column depicts the pairwise contrasts calculated between each of these conditions; thick lines represent the 50% HDI and thin lines represent the 95% HDI. Polygons depict the posterior distribution for each contrast. The proportion of know responses is estimated only for those trials not receiving a remember response (e.g., Yonelinas & Jacoby, 1995).

Because our analysis employed logistic regression, the coefficients exist in logit-space. In this metric the intercept was estimated to be −1.50 (HDI_95%_ = −1.87, −1.14) with the respective slopes for the silent and aloud contrasts being 1.58 (HDI_95%_ = 1.25, 1.93) and 2.19 (HDI_95%_ = 1.80, 2.60). To ease consumption of our results, the posterior distribution of our model was used to produce estimates for each condition that were then back-transformed into the proportion of old responses as depicted in the top panel of Figure 1. The left frame depicts the back-transformed means for each condition. The right frame depicts a violin plot of the posterior distributions for the comparisons between each of our conditions (based upon the back-transformed values); these graphical comparisons may be interpreted directly. The point in the centre of each polygon represents the (median) point estimate of that difference, the thick lines radiating from this point represent the 50% HDI and the thinner lines represent the 95% HDI. The polygons themselves depict the complete posterior distribution both above the point and also mirrored below the point. Based upon the data provided in the top panel of Figure 1, it is clear that participants were capable of discriminating either silent or aloud study items from foils—and also that they demonstrated superior recognition for aloud items relative to silent items. Having established a production effect, we next applied the same multilevel logistic model to the remember and know responses.[Fig-anchor fig1]

#### Remember responses

For the remember responses, the intercept was estimated to be −3.72 (HDI_95%_ = −4.22, −3.28) with the respective slopes for silent and aloud conditions being 2.31 (HDI_95%_ = 1.89, 2.77) and 3.10 (HDI_95%_ = 2.73, 3.49). These values were again back-transformed into the proportion of remember responses and depicted in the middle row of Figure 1. All comparisons were credibly greater than zero, demonstrating that participants were more likely to correctly recollect items they had read silently or aloud than they were to falsely recollect foil items, and also that production improved recollection relative to silent reading.

#### Know responses

Our final analysis explored how production influenced the proportion of know responses. One issue faced when analysing these particular data was the dependency between know and remember responses resulting from the fact that as the frequency of one judgment increases, there is less opportunity for the other judgment to be made. That is, in the standard remember-know procedure, participants are instructed to identify an item as remembered if they can recollect details pertaining to having studied that item, and to respond know only if recollection fails. Because recollection takes precedence, cases where an item is both familiar and recollected will receive only a remember response. For this reason, dual-process theorists often argue that the analysis of raw know responses is liable to underestimate familiarity (see Yonelinas, 2002). To address this concern, we adopted the independence remember-know method, in which familiarity is estimated by dividing the proportion of know responses by the proportion of trials for which a remember response was not made (e.g., Jacoby, Yonelinas, & Jennings, 1997; Mangels, Picton, & Craik, 2001; Ochsner, 2000; Ozubko, Gopie, MacLeod, 2012; Yonelinas & Jacoby, 1995). To achieve a similar end without first aggregating responses into proportions, we applied our logistic model after excluding trials for which a remember response had been made.^2^

Within this new model, the intercept was estimated to be −1.70 (HDI_95%_ = −2.09, −1.30) with the respective slopes for the silent and aloud conditions being 1.18 (HDI_95%_ = 0.88, 1.49) and 1.45 (HDI_95%_ = 1.09, 1.80). The back-transformed proportion of know responses are depicted in the bottom row of Figure 1. Once again, all comparisons were credibly greater than zero (the aloud − silent difference was small but still excluded 0, with a back-transformed median difference of .064, HDI_95%_ = .01, .13). Thus participants were more likely to correctly know that an item had been read silently or aloud at study than they were to falsely know that a foil had been studied, and production increased familiarity of those items relative to silent items. Replicating Ozubko, Gopie, & MacLeod (2012) then, we observed both a recollection and familiarity advantage for produced words in a within-subject design.

## Experiment 1b: Between-Subject Design With Remember-Know Judgments

Having established that the within-subject production effect is observed for both recollection and familiarity we next explored our claim that manipulating production between-subjects would result in a production effect only for know judgments. To accomplish this we replicated the methods from Experiment 1a with the exception that production was manipulated between-subjects, meaning that participants either read silently or read aloud *all* study items.

### Method

#### Participants

A sample of 37 participants enrolled at Dalhousie University took part in this experiment in exchange for partial course credit. Participants were randomly assigned to either the silent (*N* = 18) or aloud (*N* = 19) condition.

#### Stimuli and apparatus

The stimuli and apparatus were identical to those used in Experiment 1a.

#### Procedure

The procedure was identical to that used in Experiment 1a, with the exception that production was manipulated between-subjects. Although font colour was still randomized in the same manner as in Experiment 1a during the study phase, participants were instructed that the colour was meaningless and that they should either read silently or read aloud all items as per their assigned condition.

### Results and Discussion

#### Old responses

We first analysed the probability of participants scoring a hit or false alarm by collapsing remember and know responses into a single binary response. Because the between-subjects design resulted in item type (foil, study) being crossed fully with production (silent, aloud), these data were submitted to a modified version of the preceding multilevel logistic regression model that included item type, production and their interaction term as fixed-effect coefficients. We further adapted our coding to mathematically centre item type such that it was −0.5 for foil items and 0.5 for study items (as opposed to the usual 0 and 1). In doing so, we were able to calculate metrics comparable to measures of response bias and sensitivity within a broader signal detection framework (see Wright et al., 2009; Wright & London, 2009).

Within a signal detection framework, response bias refers to the propensity to say “old” irrespective of whether the item was old or new and is commonly calculated by applying separately a probit transformation (i.e., the inverse of the cumulative distribution function of the standard normal distribution) to the proportion of hits and false alarms within a given condition, and then taking their average (this produces *C*; Macmillan & Creelman, 2005). In the context of the present model this same value can be estimated by aggregating only those coefficients *not including* item type (the intercept in the case of silent items and the intercept + the coefficient for our production variable in the case of aloud items). Because these coefficients represent the propensity to say “old” irrespective of whether the item was old or new (as indicated by the exclusion of the item type variable and its interaction) their combination produces a metric similar to *C*, only on the logit (i.e., log-odds) as opposed to probit scale (we denote the scale of our measure by referring to it as *C*_*L*_).^3^ Positive values of *C*_*L*_ indicate a liberal bias (tendency to say the item was “old”) and negative values indicate a conservative bias (tendency to say the item was “new”).

Sensitivity refers to the propensity to discriminate between the old (i.e., studied) and new (i.e., foil) test items and is commonly calculated by applying separately a probit transformation to the proportion of hits and false alarms within a given condition, and then subtracting the transformed false alarms from the transformed hits (this produces *d’*; Macmillan & Creelman, 2005). In the context of the present model this same value can be estimated by aggregating only those coefficients *including* item type (the coefficient for item type in the case of silent items and the coefficient for Item Type + the Item Type × Production interaction in the case of aloud items). Because the main effect of item type and its interaction represent changes in the propensity to say “old” that are specifically related to whether the items in question had been studied previously, they may be interpreted as reflecting the degree to which participants are able to discriminate between the old and new items. Specifically, the main effect of item type would represent the logit-transformed difference between hits and false alarms for the silent condition and is therefore analogous to traditional measures of sensitivity; the Item Type × Production interaction would then represent the difference in sensitivity between the silent and aloud conditions. Together, these coefficients can be used to calculate a metric similar to *d’* for each group, but again on the logit as opposed to probit scale (we denote the scale of our measure by referring to it as *d’*_*L*_).

**Figure 2 fig2:**
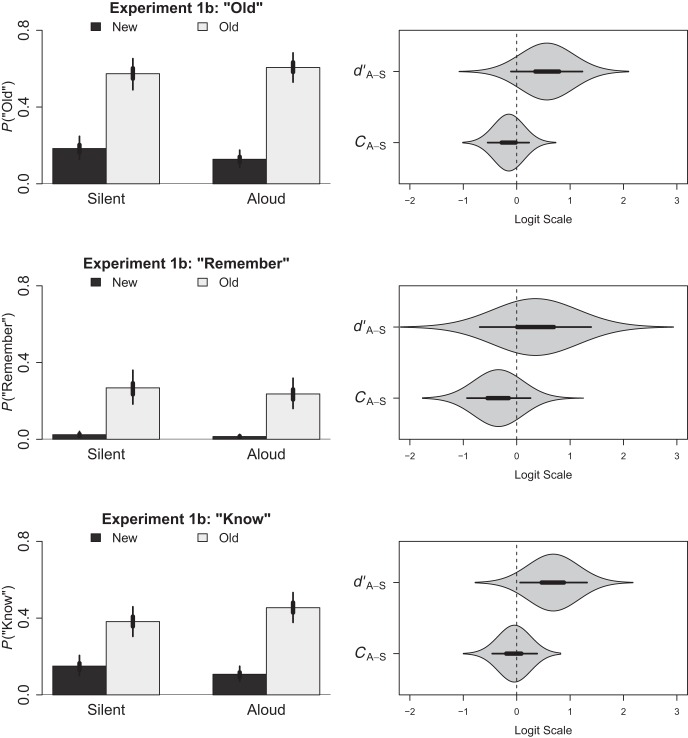
The left column depicts the back-transformed estimated proportion of old, remember and know responses for Experiment 1b as a function of production (silent, aloud) and item type (foil, target). The right column depicts contrasts comparing sensitivity (*d*_*L*_’) and response bias (*C*_*L*_; both on the logit scale, see in-text for details) as a function of production (silent, aloud); thick lines represent the 50% HDI and thin lines represent the 95% HDI. Polygons depict the posterior distribution for each contrast. The proportion of know responses is estimated only for those trials not receiving a remember response (e.g., Yonelinas & Jacoby, 1995).

In presenting the results of our signal detection model, we have chosen to report each of the coefficients for the sake of completeness. The intercept was estimated to be −0.60 (HDI_95%_ = −0.89, −0.32) and the respective slopes for production and item type were −0.15 (HDI_95%_ = −0.54, 0.23) and 1.79 (HDI_95%_ = 1.30, 2.26). The slope of the interaction term was 0.56 (HDI_95%_ = −0.10, 1.23). However, we were instead interested in the values of *C*_*L*_ and *d’*_*L*_ that can be derived from these coefficients. Therefore, the posterior distribution of our model was used to calculate estimates of *C*_*L*_ and *d’*_*L*_ for each condition (as well as the back-transformed proportion of old responses) and the relevant contrasts are depicted in the top panel of Figure 2. Based upon the statistical comparisons presented in this figure, there was no evidence that production influenced response bias. Participants demonstrated a similarly conservative response bias in both the aloud (*M* = −0.75; HDI_95%_ = −1.02, −0.47) and the silent groups (*M* = −0.60; HDI_95%_ = −0.89, −0.32). Critically, participants were capable of discriminating the study items from foils in both the aloud (*M* = 2.35; HDI_95%_ = 1.89, 2.83) and silent groups (*M* = 1.79; HDI_95%_ = 1.30, 2.26), but production did not improve this ability, even though the effect was in the predicted direction. Regardless, our main interest was not in the overall response patterns, but how recollection and familiarity contribute differentially to the between-subjects production effect. Hence, we turn now to separate analyses of recollection and familiarity.[Fig-anchor fig2]

#### Remember responses

For our analysis of the remember responses, we used the same model as in the preceding analyses including calculation of the signal detection metrics of response bias and sensitivity. A similar interpretation avails itself: Changes in response bias indicate changes in the overall tendency to make a remember response whereas changes in sensitivity indicate changes in the degree to which those remember responses differentiated study items and foil items. Within this model, the intercept was −2.36 (HDI_95%_ = −2.78, −1.93) and the respective slopes for production and item type were −0.34 (HDI_95%_ = −0.93, 0.26) and 2.70 (HDI_95%_ = 1.96, 3.42). The slope of the interaction term was 0.35 (HDI_95%_ = −0.69, 1.39). However, as before our hypotheses dealt with changes in *C*_*L*_ and *d’*_*L*_ rather than the model coefficients themselves, and the relevant statistical contrasts are therefore depicted in the middle panel of Figure 2. There was no evidence that production influenced response bias. Instead, participants demonstrated a substantially conservative response bias in both the aloud group (*M* = −2.70; HDI_95%_ = −3.14, −2.27) and the silent group (*M* = −2.36; HDI_95%_ = −2.78, −1.93). However, participants nonetheless discriminated the study items from foils in both the aloud group (*M* = 3.05; HDI_95%_ = 2.31, 3.84) and silent group (*M* = 2.70; HDI_95%_ = 1.96, 3.43). There was no evidence that production improved this ability.

To the extent that the effect of production on recollection is aligned with the use of a distinctiveness-based strategy at test the absence of a between-subjects production effect for recollection could be viewed as novel support for MacLeod et al.’s (2010) contention that such a strategy is either overlooked or ineffective in between-subjects designs. Our findings likewise echo the failure to observe a between-subjects production effect in studies using recall as their dependent measure (e.g., Jones & Pyc, 2014)—which might also rely on recollection.

#### Know responses

To evaluate whether our between-subjects production manipulation influenced the familiarity of the studied items, we next fit an analogous model to the know responses, excluding those trials for which remember responses were made as in Experiment 1a. Within this model, the intercept was −1.11 (*HDI*_*95%*_ = −1.42, −0.81) and the respective slopes for production and item type were −0.04 (HDI_95%_ = −0.46, 0.38) and 1.25 (HDI_95%_ = 0.80, 1.70). The slope of the interaction term was 0.68 *(HDI*_*95%*_ = 0.07, 1.32). Estimates of *C*_*L*_ and *d’*_*L*_ for each condition were calculated and are depicted alongside the relevant contrasts in the bottom panel of Figure 2. Participants again demonstrated a conservative response bias in both the aloud group (*M* = −1.15; HDI_95%_ = −1.45, −0.86) and the silent group (*M* = −1.11; HDI_95%_ = −1.42, −0.81). However, in the current case participants were not only capable of discriminating the study items from foils in both the aloud group (*M* = 1.94; HDI_95%_ = 1.50, 2.38) and silent group (*M* = 1.25; HDI_95%_ = 0.80, 1.70), but the contrasts depicted in Figure 2 also demonstrate that production improved discrimination.

In sum, reading a word aloud improved familiarity to a greater degree than reading a word silently, casting new light on past concerns regarding the diminutive nature of the between-subjects production effect in recognition (see Fawcett, 2013). Our present findings provide the first evidence that production enhances familiarity when manipulated between-subjects, but does not impact recollection—supporting our dual-process account of the production effect in recognition memory.

## Experiment 2a: Within-Subject Design With Confidence Ratings

Using the remember-know paradigm, Experiments 1a–b established that production increases both recollection and familiarity when manipulated within-subjects, but increases only familiarity when manipulated between-subjects. These findings replicate earlier work (Ozubko et al., 2012) whilst imposing new and important boundary conditions on the effect, potentially explaining why the between-subjects production effect is less reliable. Experiment 2a followed Ozubko et al. (2012) by attempting to replicate this pattern using a different methodological and analytical framework. To this end, our remaining experiments instead adopted a dual-process signal detection approach in which familiarity and recollection could be inferred covertly on the basis of confidence ratings (Yonelinas, 1994, 1997, 2001).

### Method

#### Participants

A sample of 25 participants enrolled at Dalhousie University took part in this experiment in exchange for partial course credit.

#### Stimuli and apparatus

The stimuli and apparatus were identical to those used in Experiment 1a.

#### Procedure

The procedure was identical to that used in Experiment 1a with the exception that during the test phase, participants did not make a remember-know judgment. Rather, they were instead presented with a scale ranging from 1 (*absolutely sure new*) to 6 (*absolutely sure old*) and rated how confident they were that the current test item had been studied. Specifically, participants were instructed to respond with the numbers 1, 2, or 3 to indicate that they were *absolutely*, *very* or *somewhat sure* that the item was new or to respond with the numbers 4, 5, or 6 to indicate that they were *somewhat*, *very* or *absolutely sure* that the item was old. A scale indicating the value of each response was provided at the bottom of the screen. Following Ozubko et al. (2012), participants were asked to use each of the numbers on the scale at some point during the test phase.

### Results and Discussion

Hit rates were initially plotted against false alarm rates at different levels of confidence to estimate the ROC curve for each subject. A dual-process signal detection model was then used to compute estimates of recollection and familiarity based on the shape and position of each curve (Yonelinas, 1994, 1997, 2001). The relation between this approach and the remember-know judgments employed in Experiments 1a–b (as well as the underlying assumptions) is discussed at length elsewhere (see Ozubko et al., 2012). In the current case, ROC curves were estimated using an optimization algorithm within the *R* programming language implementing the same solution used by Yonelinas’ dual-process signal detection (DPSD) solver (available from http://psychology.ucdavis.edu/Labs/Yonelinas/PWT/). To keep the subsequent analyses and discussion focused, the ROC curves are depicted in the Supplementary Online Materials.

#### Old responses

**Figure 3 fig3:**
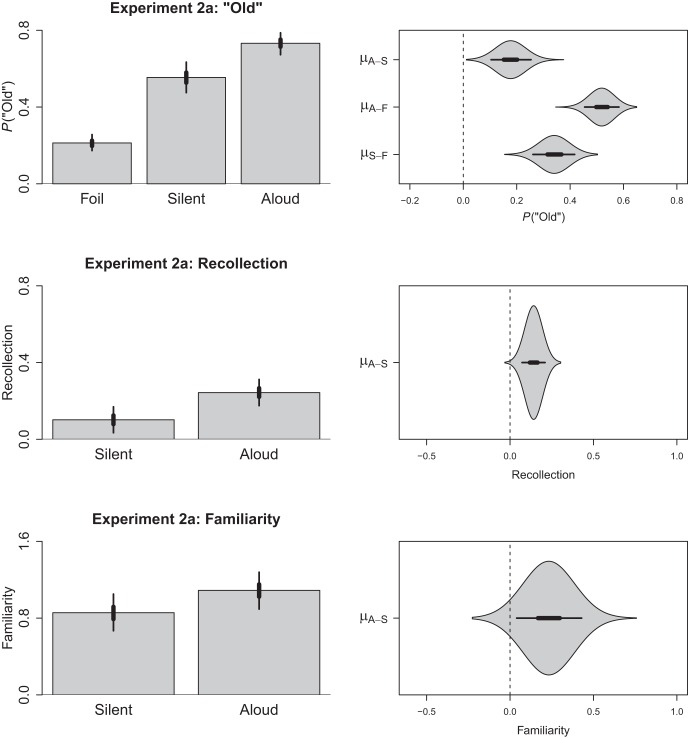
The left column depicts the predicted proportion of old responses and estimates of recollection and familiarity for Experiment 2a as a function of item type (foil, silent, aloud) or production (silent, aloud). The right column depicts the pairwise contrasts calculated between each of these conditions; thick lines represent the 50% HDI and thin lines represent the 95% HDI. Polygons depict the posterior distribution for each contrast. Each row is on a different scale.

Although not of primary interest, in keeping with earlier experiments we initially converted our confidence ratings into binary responses by rescoring each 1, 2, or 3 response as new and each 4, 5, or 6 response as old. The resulting data were then submitted to the model used in Experiment 1a. The intercept was estimated to be −1.31 (HDI_95%_ = −1.56, −1.06) and with the respective slopes for silent and aloud being 1.53 (HDI_95%_ = 1.18, 1.87) and 2.31 (HDI_95%_ = 1.97, 2.67). The back-transformed proportion of old responses is depicted in the top row of Figure 3 alongside the relevant contrasts, which demonstrate a credible production effect.[Fig-anchor fig3]

#### Recollection

Because our estimation procedure produced only a single estimate of recollection for the aloud and silent conditions for each subject, these data were submitted to a Gaussian regression model with production (silent, aloud) treated as a fixed effect. Because production was a categorical variable, it was dummy coded (i.e., silent = 0, aloud = 1) such that the intercept represented recollection for the silent condition and the slope represented the difference in recollection between the silent and aloud conditions. Within this model, the intercept (i.e., performance in the silent condition) was .10 (HDI_95%_ = .03, .17) and the difference between the production conditions was .14 (HDI_95%_ = .07, .21). In short, as depicted in the middle panel of Figure 3, this finding replicates the effect of production on recollection observed in Experiment 1a.

#### Familiarity

The same general pattern emerged for the familiarity estimates, for which the intercept (i.e., performance in the silent condition) was 0.86 (HDI_95%_ = 0.67, 1.05) and the difference between the production conditions was 0.23 (HDI_95%_ = 0.04, 0.43). These data are depicted in the bottom panel of Figure 3. This finding also replicates the effect of production on familiarity observed in Experiment 1a. Hence, both recollection and familiarity were found to support the production effect in the within-subject design used in Experiment 2a.

## Experiment 2b: Between-Subject Design With Confidence Ratings

Experiment 2b explored our central hypothesis that manipulating production between-subjects would result in a production effect only for estimates of familiarity. To accomplish this we replicated Experiment 2a with the exception that production was now manipulated between-subjects.

### Method

#### Participants

A sample of 44 participants enrolled at Dalhousie University took part in this experiment in exchange for partial course credit. Participants were randomly assigned to either the read silently (*N* = 22) or read aloud condition (*N* = 22).

#### Stimuli and apparatus

The stimuli and apparatus were identical to those used in Experiment 2a.

#### Procedure

The procedure was identical to that used in Experiment 2a, except that production was manipulated between-subjects.

### Results and Discussion

Confidence ratings were fit using the DPSD model to compute estimates of recollection and familiarity for the aloud and silent items on a subject-by-subject basis as in Experiment 2a. ROC curves are again depicted in the Supplementary Online Materials.

#### Old responses

**Figure 4 fig4:**
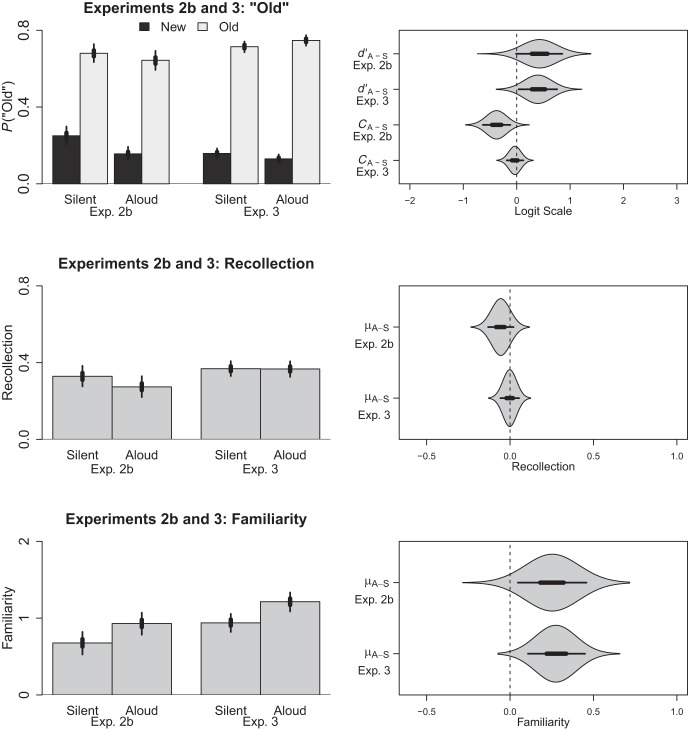
The left column depicts the predicted proportion of old responses and estimates of recollection and familiarity for Experiments 2b and 3 as a function of item type (foil, target) and/or production (silent, aloud). The right column depicts the pairwise contrasts comparing sensitivity (*d*_*L*_’), response bias (*C*_*L*_; both on the logit scale, see in-text for details), recollection or familiarity between each of these conditions; thick lines represent the 50% HDI and thin lines represent the 95% HDI. Polygons depict the posterior distribution for each contrast. Each row is on a different scale.

The probability of making an old response was analysed using the model described for Experiment 1b. In this model, the intercept was estimated to be −0.17 (HDI_95%_ = −0.36, 0.00) and the respective slopes for production and item type were −0.38 (HDI_95%_ = −0.63, −0.12) and 1.85 (HDI_95%_ = 1.56, 2.16). The slope of the interaction term was 0.43 (HDI_95%_ = −0.01, 0.85). This time the contrasts depicted in Figure 4 revealed differences between the silent and aloud groups: Whereas participants in the aloud condition were more conservative (*C*_*L*_ = −0.55, HDI_95%_ = −0.73, −0.36) compared with the silent condition (*C*_*L*_ = −0.55, HDI_95%_ = −0.73, −0.36), there was a tendency for participants to be better at discrimination in the aloud condition (*d’*_*L*_ = 2.28, HDI_95%_ = 1.97, 2.59) compared with the silent condition (*d’*_*L*_ = 1.85, HDI_95%_ = 1.56, 2.16). Therefore, unlike in Experiment 1b, the present data revealed evidence of a credible between-subjects production effect even as measured by old responses. Nonetheless, we were primarily interested in how production influenced estimates of recollection and familiarity.[Fig-anchor fig4]

#### Recollection

Because our estimation procedure produced now only a single estimate of recollection for the aloud *or* silent items for each subject, our models became between subject in nature. Otherwise, they were identical to those used in Experiment 2a. Within this model, the intercept was .33 (HDI_95%_ = .28, .38) and the difference between the production groups was −.06 (HDI_95%_ = −.13, .02). In short, as depicted in the middle panel of Figure 4, this finding replicates the apparent absence of an effect of production on recollection observed in Experiment 1b. If anything, the difference was in the opposite direction (i.e., a reverse production effect).

#### Familiarity

An identical model was applied to the familiarity estimates. Within this model the intercept was 0.68 (HDI_95%_ = 0.53, 0.82) and the difference between the production conditions was 0.25 (HDI_95%_ = 0.05, 0.46). These data are depicted in the bottom panel of Figure 4, and replicate the effect of production on familiarity observed in Experiment 1b.

## Experiment 3: Web-Based Between-Subject Design With Confidence Ratings

Having established that production affects both recollection and familiarity when manipulated within-subjects, but affects only familiarity when manipulated between-subjects, we next provided a replication of the between-subjects pattern using a larger sample. To achieve this, we implemented our task as a web application. We chose to replicate the method of Experiment 2b rather than Experiment 1b because confidence ratings are easier to explain than remember-know ratings via written instructions.

Experiment 3 also examined a methodological issue regarding design effects in the production literature. Namely, participants in a typical within-subjects production experiment read aloud half as many words as a participant in a pure aloud condition of an otherwise matched between-subjects production experiment. Rather than representing differences in the underlying mechanisms involved, design effects could arise as a result of this confound. To investigate this possibility, Experiment 3 varied the number of words in the aloud condition and also included filler trials to make conditions more comparable to our within-subjects condition. The details of these manipulations are presented primarily in the Supplementary Online Materials, but to summarise their outcome, the number or spacing of the words read aloud did not appear to affect the magnitude of the production effect.

### Method

#### Participants

A total of 369 students enrolled at University of Toronto Scarborough signed up to participate online in exchange for partial course credit of which 184 participated in the aloud condition and 185 participated in the silent condition. The data were screened to exclude participants who did not complete the entire task, took part more than once or reported any extraexperimental activities that might have influenced their performance (e.g., speaking with a friend, taking a midexperiment break). These exclusion criteria resulted in 269 usable participants (129 in the aloud condition, 140 in the silently condition).

#### Stimuli and apparatus

The same list of 240 words was used again. However, words and all instructions were presented via the participants’ web-browser in lowercase 14-point font.

#### Procedure

##### Study phase

After providing informed consent, participants were provided with instructions detailing the study phase. It was also our intention to manipulate the spacing between the trials in this experiment. However, this manipulation failed to produce any compelling effects or interactions, so for the sake of exposition we discuss the methodological differences between these conditions below but otherwise collapse them into read aloud and read silently conditions for the purpose of analysis and interpretation. Further details, including analyses taking our spacing manipulation into account are provided in the Supplementary Online Materials.

This experiment was conceived as a 2 (Production: aloud, silent) × 3 (Spacing: standard, filler, short) between-subjects design producing six conditions in total. The instructions for the production manipulation were identical to the preceding experiments. For the spacing manipulation, in the standard or short condition half of the words were presented in green and half were presented in purple, although participants were told to ignore the colour. In the standard condition participants studied 120 words, whereas in the short condition participants studied only 60 words. This manipulation matched the number of words read aloud in the short condition to the number of words read aloud in our within-subject experiments. The filler condition was identical to the short condition, except all the words were presented in the same colour (i.e., either green or purple) and 60 filler items (i.e., XXXXX’s) matched for word length and presented in the opposite colour were randomly interspersed amongst the word trials. The purpose of the filler condition was to match the overall study list length with the standard condition while reducing the number of items read aloud to match our earlier within-subject experiments. Participants were told that filler trials would occur throughout the study phase, and could be ignored.

Below each item during the study phase was a “Next” button that became active after 2 s and when clicked proceeded to the next trial. Each trial began with a fixation cross (“+”) presented for 500 ms and an intertrial interval (intertribal interval [ITI]) of 500 ms was used. The words for each individual participant were drawn randomly from the full set of 240 words, as was the colour assignment (i.e., which stimulus set would be green and which would be purple).

##### Test phase

At test, words appeared individually in a black font, and participants had to identify whether the word was studied (i.e., old) or new. Participants made their choice by clicking a value on a 6-point scale, ranging from 1 (*sure new*) to 6 (*sure old*), shown directly below each word at test. Participants were encouraged to use the entire scale over the course of the test, and to avoid strategies that would result in binary response data, such as selecting 6 and 1 or 5 and 2 for all their responses. In the standard spacing condition, the 120 studied words were randomly intermixed with 120 new words. In the short and filler conditions, the 60 studied words were randomly intermixed with 60 new words, randomly drawn from the full word pool. Each trial began with a fixation cross that was presented for 500 ms, and a 500 ms ITI was used between trials. Following completion of the test phase, participants once again completed a strategy questionnaire, which is discussed in the Supplementary Online Materials.

### Results and Discussion

The analyses were identical to Experiment 2b and the ROC curves are presented in the Supplementary Online Materials.

#### Old responses

The probability of participants labelling an item as “old” was analysed using the logistic model described in Experiment 2b. For this model, the intercept was estimated to be −0.38 (HDI_95%_ = −0.48, 0.27) and the respective slopes for production and item type were −0.03 (HDI_95%_ = −0.19, 0.12) and 2.59 (HDI_95%_ = 2.35, 2.84). The interaction term was 0.40 (HDI_95%_ = 0.04, 0.76). The contrasts depicted in the top panel of Figure 4 demonstrate a conservative response bias for both the aloud group (*C*_*L*_ = −0.41, HDI_95%_ = −0.52, −0.30) and the silent group (*C*_*L*_ = −0.38, HDI_95%_ = −0.48, −0.27). Nonetheless, participants demonstrated better discrimination in the aloud group (*d’*_*L*_ = 2.97, HDI_95%_ = 2.73, 3.25) than in the silent group (*d’*_*L*_ = 2.59, HDI_95%_ = 2.35, 2.84) supporting the presence of a between-subjects production effect.

#### Recollection

For the analysis of recollection, the intercept was .37 (HDI_95%_ = .33, .41) and the difference between the production groups was .00 (HDI_95%_ = −.06, .05). As depicted in the middle panel of Figure 4, there was no evidence of an effect of production on recollection in the context of a between-subjects design. In fact, the present effect was centered at 0 with 52.47% of credible values below 0 and 47.53% of credible values above 0 with fully half of the credible values concentrated between −.02 and .02.

#### Familiarity

In contrast to the analysis of recollection, when the same model was applied to familiarity a credible difference was observed. This difference is depicted in the bottom panel of Figure 4. Within this model, the intercept was 0.93 (HDI_95%_ = 0.82, 1.06) and the difference between the production groups was 0.28 (HDI_95%_ = 0.11, 0.45). In short, we replicated the effect of production on familiarity when manipulated between-subjects.

## Meta-Analysis

Across five experiments we have shown that whereas the within-subject production effect is driven by both recollection and familiarity, the between-subjects production effect is driven by familiarity alone. Before turning to a critical discussion of our results, we present a meta-analytic synthesis of the presently reported data along with: (a) the experiments conducted by Ozubko et al. (2012), and, (b) an unpublished pilot study conducted by a separate research group with methods similar to Experiment 1a (*N* = 35; Roddick, Fawcett, Newman, Lambert & Bodner, 2014).^4^

**Figure 5 fig5:**
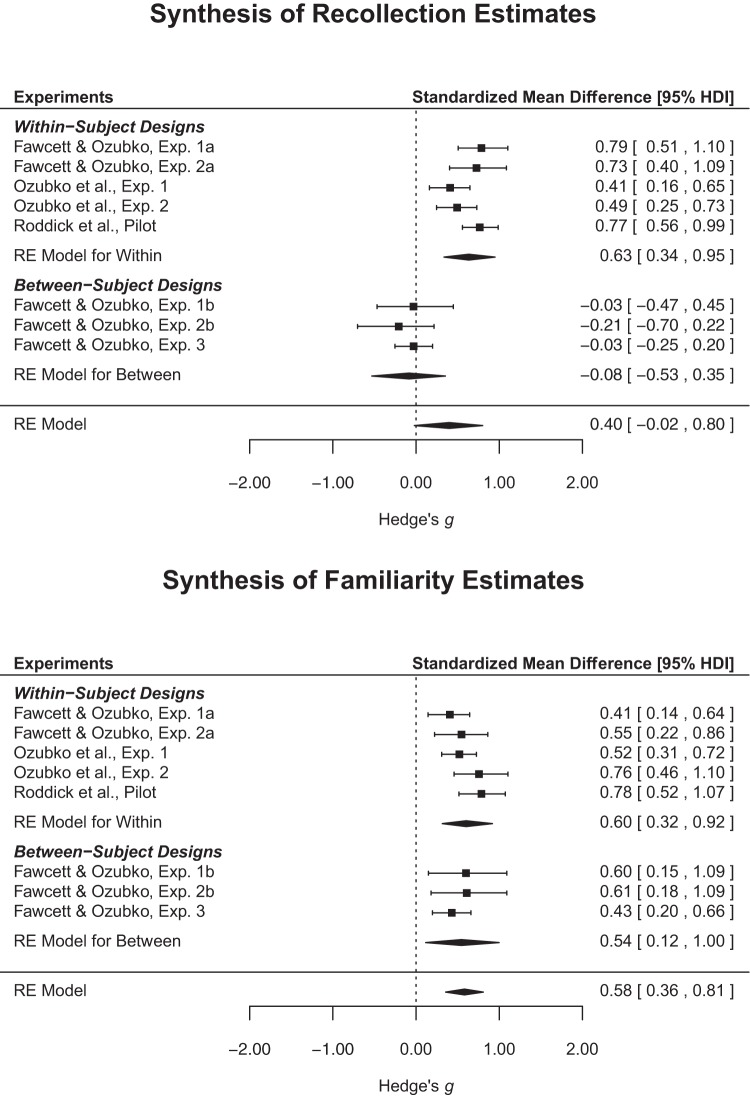
Forest plots aggregating recollection (top) and familiarity (bottom) estimates from the current experiments as well as those reported by Ozubko et al. (2012) and an unpublished study by Roddick et al. (2014). Effect sizes were submitted to separate Bayesian models: Polygons at the bottom of each plot represent the median effect (and 95% HDI) estimated from a model intermixing the within- and between-subjects experiments; the remaining polygons represent the median effects (and 95% HDIs) estimated from a model incorporating study design (within, between) as a moderator. Raw effect sizes are provided in the Supplementary Online Materials.

For each set of data, we calculated separate effect sizes (Hedges’ *g*; Hedges, 1982) using a custom script implemented within *R 3.1.1* (R Core Team, 2014); within-subject effects were calculated using the appropriate “raw score” metric to equate them with the between-subjects effects (Morris & DeShon, 2002). Remember-know judgments (current Experiments 1a and 1b; Ozubko et al., 2012, Experiment 1; Roddick et al., 2014) were first converted into *d’* scores (after following the independence remember-know procedures describe above; e.g., Jacoby et al., 1997); dual-process signal detection estimates of familiarity and recollection (Ozubko et al., 2012, Experiment 2; current Experiments 2a–b and 3) were submit to the effect size calculations directly. Once calculated, effect sizes were then analysed using separate Bayesian meta-analytic models depicted in the forest plots provided in Figure 5. Two models were employed for each measurement: The first was a basic random-effects model with the second expanding upon this model to include study design as a moderator. Fixed-effects models produced identical (albeit less conservative) outcomes.[Fig-anchor fig5]

The findings depicted in Figure 5 nicely summarise the major conclusions of the present experiments. Whereas the overall estimate for measures of recollection did not credibly differ from 0, there was a moderate amount of observed heterogeneity (τ = 0.46; HDI_95%_ = 0.18, 0.92). The source of this heterogeneity is readily established through inspection of the effects themselves. The within-subject experiments produced consistently larger effect sizes than the between-subjects experiments (within-between = 0.72; HDI_95%_ = 0.20, 1.27), which hovered slightly below 0 (representing a tendency toward a reverse effect). After accounting for study design, a measurable amount of heterogeneity remained in the regression model (τ = 0.23; HDI_95%_ = 0.03, 0.55), but study design nonetheless accounted for approximately 51.58% of the variability observed in the initial model (calculated as [0.46–0.23]/0.46).

Analysis of familiarity estimates produced no evidence of design effects. As revealed in the bottom panel of Figure 5, the effects demonstrated surprising consistency irrespective of study design. Supporting this evaluation, the basic model demonstrated a similar degree of heterogeneity (τ = 0.20; HDI_95%_ = 0.03, 0.45) relative to the regression model (τ = 0.23; HDI_95%_ = 0.04, 0.53), with the contrast between designs balanced at −0.05 (HDI_95%_ = −0.58, 0.48). Having established the consistency of the design effects across the available literature on this topic, we next turn to the theoretical implications.

## General Discussion

Though the production effect was originally described as a within-subject phenomenon, recent evidence has demonstrated a reliable albeit small between-subjects effect (Fawcett, 2013). By considering how recollection and familiarity are influenced by production, we were able to reconcile the role of distinctiveness with the between-subjects production effect. Experiments 1 and 2 used converging measures of recollection and familiarity and supported past findings that the within-subject production effect arises due to advantages in both (e.g., Ozubko et al., 2012). Importantly, however, in three experiments we also found a between-subjects production effect observed *only* for estimates of familiarity and not for recollection. In fact, the magnitude of the production effect observed for familiarity was surprisingly consistent regardless of study design (see Figure 5). These findings support a dual-process account of production and clarify why some past studies failed to observe a significant between-subjects production effect: the between-subjects production effect lacks a recollective component.

### A Dual Process Interpretation: Memory Strength and Relative Distinctiveness

In the introduction we predicted that the strategic use of distinctive information at test was a recollective phenomenon and would occur only within-subjects, whereas the influence of production on familiarity would operate both within- and between-subjects. The fact that this dual-process account was supported challenges the current theoretical explanation for the production effect in recognition memory. The production effect has often been attributed predominantly to a distinctiveness-based strategy at test whereby participants use the availability of a recent production trace (i.e., memory of having produced the item) to discriminate between old and new items (MacLeod et al., 2010). This account has received much support, including the finding that the production effect can be eliminated by having participants produce the foil items prior to study (Ozubko & MacLeod, 2010; cf., Bodner & Taikh, 2012), and is reduced in populations known to experience difficulty with distinctive encoding (e.g., older adults; Lin & MacLeod, 2012). However, our findings suggest that multiple mechanisms can generate a production effect. Specifically, we argue that production enhances memory not only through the inclusion of distinctive information, such as motor movements or auditory details related to having said the word (i.e., the production trace), but also by strengthening the representation of the produced items. We speculate that whereas the former process (relative distinctiveness) is represented via recollection in our within-subject but not between-subjects manipulations, the latter process (memory strength) is indexed by enhancements to familiarity in both our within-subject and between-subjects manipulations.

Accepting that production improves the strength of a representation in memory, it remains unclear as to why this would be the case. One possibility is that the relationship between production and memory strength is mediated in part by the amount of attention participants dedicate to the produced items. This idea is supported by the fact that even the intention to produce an item (prior to the actual productive act) modulates the magnitude of electrophysiological markers of attentional engagement and distinctive processing (i.e., the P300; Hassall, Quinlan, Turk, Taylor, & Krigolson, submitted). Similarly, our participants reported paying less attention to nonproduced items (e.g., see the Supplementary Online Materials) and previous research has observed more mind-wandering whilst participants read passages silently than when reading them aloud (Varao Sousa, Carriere, & Smilek, 2013). In a real-world setting, production in the form of note taking during a classroom lecture not only predicted attentional engagement but also academic performance in the course (Lindquist & McLean, 2011). Critically, engagement with the course material was a better predictor of learning outcomes than production itself. On the basis of these findings, we speculate that the familiarity-based component of the production effect may be driven partially by constructs such as task engagement, although further research is required to evaluate this possibility.

However, our dual-process account is not without challenge. As noted earlier, the production effect is often attributed to a distinctiveness-based strategy at test (“I remember saying it aloud so I must have studied it”). However, participants in our between-subjects experiments commonly reported using this strategy when responding to test items (see the Supplementary Online Materials). Presuming these retrospective reports are accurate, the fact that participants use productive information at test regardless of study design begs the question as to why the production effect is absent for measures of recollection in between-subjects designs. We do not yet have a decisive answer to this challenge, though online strategy judgments would prove useful in determining what precisely participants are doing at test. Another possible explanation comes from recent modelling work by Elfman, Parks, and Yonelinas (2008) who argued that the hippocampus—a structure critical for recollection—loses its ability to encode items distinctively as the level of feature similarity across items increases. In other words, recollection itself may begin to break down in situations where stimuli are too similar to one another. In terms of the production effect, producing every item (as done in a between-subjects design) may cause encoding of the distinctive elements of the produced items to fail, rendering a distinctiveness-based recollection strategy ineffective.

### A Single-Process Interpretation: Only Memory Strength

Although a wealth of psychological and neuroscientific evidence supports the view that recollection and familiarity represent qualitatively unique memory processes (Eichenbaum et al., 2007; Perfect & Dasgupta, 1997; Rajaram, 1993; Skinner & Fernandes, 2007; Yonelinas, 2002), competing accounts instead view the subjective experience of recollection or familiarity as representing differences in the overall strength of the corresponding memory along a single dimension (e.g., Donaldson, 1996). It is therefore important to also consider whether a single-process account would provide a more parsimonious explanation of our results (e.g., Wixted, 2007; Wixted & Stretch, 2004).

Such a single-process account of the present findings would begin with the assumption that items vary in strength even prior to encoding (Jang, Wixted, & Huber, 2011; Mickes, Wixted, & Wais, 2007; Wixted, 2007). At study, the strength of any given item would then increase dependent upon idiosyncratic factors such as the amount of attention or rehearsal dedicated to that item. Production would then provide a further increment to the strength of the produced items. By virtue of this increment, the proportion of weak (familiarity-based) and strong (recollection-based) memories should be greater for produced items than for nonproduced items. Indeed, this is the precise pattern observed in our within-subject experiments. However, our between-subjects experiments resulted in a different pattern—with production increasing only the proportion of weak memories (familiarity-based) with no impact on the proportion of strong memories (recollection-based). Taken together, a single-process interpretation of our findings would therefore conclude that production strengthens both weakly and strongly encoded items when manipulated within-subjects, but only weakly encoded items when manipulated between-subjects. Because we can see no reason to expect that production would preferentially benefit weakly encoded items in between-subjects designs, we are presently unable to reconcile a single-process account with our data and therefore prefer the dual-process account described earlier. Nonetheless, a single-process explanation for the design effects in the present experiments may emerge in the future.

## Conclusion

In summary, our experiments show that whereas the production effect in recognition memory is supported by both recollection and familiarity in within-subject designs, it is supported by familiarity alone in between-subjects designs. We interpret these results in the context of a dual-process account of the production effect, which attributes the effect of production to differences in relative distinctiveness (as indexed by recollection) and differences in memory strength (as indexed by familiarity). This novel finding may explain why a significant between-subjects production effect has not always been found, and may lead to new ways of thinking about how production influences memory.

## Supplementary Material

10.1037/cep0000089.supp

## Footnotes

1 Two words (*time* and *inhabitant*) were excluded from this calculation in the former case because the frequency was extreme (i.e., 1,599) and in the latter case because it did not have a corresponding Kučera-Francis word frequency. Excluding either or both from analyses had no impact upon study outcomes.

2 The validity of logistic regression as a means of approximating the independence remember-know procedure rests upon the fact that dividing the proportion of know responses by the proportion of nonremember trials (see Ozubko et al., 2012, p. 328) is equivalent mathematically to calculating the proportion of know responses after excluding trials for which a remember response was made. With this in mind, a logistic regression model applied to the data for nonremember trials is simply a more flexible method of estimating the same population parameter. Adoption of this approach also honors the binomial nature of the data under investigation and permits hierarchical modeling or the inclusion of trial level predictors. For further discussion and proof, see Fawcett et al. (2016).

3 The fact that item type has been centered is critical to this calculation because it ensures that the intercept and coefficient for the slope of the production variable are relevant to the average of the foil and study conditions; had item type instead been coded as 0 (foil item) and 1 (study item) the intercept would instead (erroneously) correspond to the propensity to say “old” to foil items alone (i.e., logit-transformed false alarms) rather than the propensity to say “old” averaged across foil and study items.

4 Roddick et al. (2014) employed a design similar to Experiment 1a with a few minor exceptions. They incorporated an additional condition similar to the present aloud condition albeit inert and intended only to control for motor activity. That condition is not relevant to the present comparisons and was excluded. Otherwise, their timings were similar although their study and test phases included fewer trials in each (90 study phase and 120 test phase).
