# Supplementary material for: Familiarity, but not Recollection, Supports the Between-Subject Production Effect in Recognition Memory
Source: Can J Exp Psychol. 2016 Jun;70(2):99–115. doi: 10.1037/cep0000089 (PMC4886847; doi:10.1037/cep0000089)
Supplement: Supplementary file 1 [file Old_Familiar_Feeling_Online_Supplement_Final.docx]

**Those old familiar feelings:**

**Familiarity, but not recollection, supports the between-subject production effect**

**Online Supplement**

This supplement provides additional detail pertaining to the method and statistical outcomes of the experiments reported in-text. Further details are available from the first author.

**Methodological Details**

*Experimental Materials*

We have provided the complete word list used in each of our experiments in Table S1.

*Strategy Questionnaires*

Following completion of the test phase for each experiment, participants completed a strategy questionnaire. For Experiments 1 and 2, this questionnaire asked the participants: (a) to describe any strategies they may have used during the course of the study or test portion of the experiment; (b) whether at test they tried to strategically recall whether they had said any of the words aloud (and if so, why), and, (c) whether at study they found themselves paying relatively more attention to the words that had been presented in green or the words presented in purple. Each question was open-ended and their responses were typed into a prompt provided on-screen and submitted using the return key. Following Experiment 3, participants completed a modified version of this questionnaire intended to assess their strategies during the experiment, as well as to confirm that they had read and followed the instructions. As before, the questionnaire asked participants to provide a short description of the strategies they used at encoding and of the strategies they used at test. However, new to this version, participants were also asked, on a 6-point scale ranging from 1 (*never*) to 6 (*always*), to identify how often during the test they could remember that a studied word had been studied because they could also remember that it had been read aloud. Participants were then asked how frequently they relied on remembering that an item had been spoken aloud to help with their recognition decisions (on the same 6-point scale) and how much attention they paid to the green items and the purple items on a similar 6-point scale. Finally, two questions were included to verify that participants had been paying attention and following the instructions. These questions asked participants to identify whether the green and the purple items at study were words, filler items (i.e., XXXXX’s), or not present. Participants with unsatisfactory answers on these questions (i.e., incorrect or incomplete responses) were considered to have failed to complete the entire task and were therefore excluded from all supplementary or in-text analyses as discussed in the method section of Experiment 3.

*Statistical Models*

We have implemented our primary analyses as multilevel models analogous to the linear mixed-effects models discussed by Dixon (2008); although our models were implemented using the *Stan* modeling language (Stan Development Team, 2013). Except where otherwise stated, models employed the “maximal” random structure justified by the design in question (Barr, Levy, Scheepers & Tily, 2013). In this context, random structure refers to the manner in which nuisance variance (such as individual differences in memory performance and response to experimental conditions) is accounted for across participants or study items and incorporated into the model; this is analogous to the within-subject error term that is calculated in many traditional repeated-measures analyses and should not be confused with the fixed effects that correspond to our manipulations of interest. Further details pertaining to our models as implemented in the *Stan* modeling language (Stan Development Team, 2013) are available upon request from the first author (for a tutorial, see Sorensen et al., submitted).

Terms relevant to the random structure of our models are not reported here but are also available upon request from the first author. For Experiments 1a-b we initially included random intercepts and slopes corresponding to both Subject and Item. This meant including a unique intercept term for each subject and item to account for differences in the overall probability of hits or false alarms being made either by individual participants or in response to individual items; unique slope coefficients were also included for each subject and item (when justified by our design; see Barr et al., 2013) to account for differences in the degree to which specific participants or items were responsive to our manipulations. In either case, the overall distribution of each random intercept or slope was modeled as arising from a normal distribution centered on 0 with an unknown variance. Using this approach, we were able to model individual differences rather than simply adding them to our error term. Whereas the random structure corresponding to subject was always informative, the random structure corresponding to item proved to explain relatively less variability and was slow to converge. Because item could only be incorporated into our initial experiments (Experiments 2a-b and 3 dealt largely with aggregate values, so random structure by item was no longer relevant), and to make our analyses more tractable, we reverted to a random structure corresponding only to subject. Nonetheless, all models were also conducted using item parameters (as appropriate) and the results were the same. In all cases, suitably uninformative priors were placed upon the coefficients in our model and sensitivity analyses revealed our findings to be robust across a range of priors. Estimates are reported along with highest-density intervals (HDIs; Kruschke, 2010) calculated for the posterior distribution of the relevant parameter. The HDIs serve a similar function to Frequentist confidence intervals with the exception that their interpretation is more intuitive: They are a direct representation of the most credible values of the estimated parameter given the mathematical combination of the prior distribution of beliefs incorporated into our model for those parameters (which were intentionally skeptical) and the current data.

Finally, because our approach employed an iterative sampling technique known as Markov chain Monte Carlo (MCMC) sampling (for the technical details relevant to our particular implementation, see Hoffman & Gelman, 2014), it is important to ensure that sufficient iterations were included for each of our models to converge upon a single solution representing the most credible parameters. To be safe we included a very large number of iterations for each model – each representing a single, random draw from the relevant posterior distribution of our parameters. As is standard practice, each model also incorporated four independent “chains” with random starting points for each parameter. In essence, these chains represent independent replications of the model itself and are included to ensure that each converges upon the same overall solution. However, because each chain starts at a different point in the model space, it always requires some number of iterations to converge upon the optimal solution – and the precise number of iterations varies from chain to chain depending upon how far from the optimal model space that chain began. Therefore, it is important to discard the initial samples from each chain (often called the “warm-up” or “burn-in” period) to ensure that the final solution has been reached prior to sampling values intended for analysis. Presuming that each chain converges on a solution, the initial (warm-up) samples are discarded and the chains are then collapsed for analysis. With this in mind, our models were conducted using six independent chains of 8000 iterations (48000 in total) with a warm-up/burn-in period of 4000 iterations per chain (24000 total) resulting in 24000 usable samples. Convergence was tested via visual inspection of the chains and also using the R-hat statistic (in all cases R-hat ≈ 1 and N_Effective_ > 5000, indicating convergence; Gelman & Hill, 2007; also discussed on p. 511 of Kruschke, 2010).

**Supplementary Analyses**

Having described our statistical approach, we next move on to the presentation of supplementary analyses that we felt detracted from the brevity and focus of the main text. We begin with a consideration of the manipulation of spacing originally intended for Experiment 3 and then the strategy questionnaires that we included following each experiment. In the following section, we close by re-analyzing our core findings using Frequentist techniques (e.g., t-tests) of the nature often reported in the cognitive literature.

*Analysis of Spacing Effects in Experiment 3*

In addition to providing a large-sample replication of Experiment 2b it was our original intention for Experiment 3 to determine whether spacing effects or differences in the number of studied items could account for any part of the design effects observed in Experiments 1 and 2. In a typical within-subject experiment, produced items are interspersed amongst silent items. These “breaks” between each instance of production could provide an opportunity for participants to linger on the produced items (this is especially possible given that participants often self-report focusing more on those items) or could otherwise facilitate the encoding of distinctive information – for example, by providing more time for the processing of that information. Further, our between-subject experiments included the same number of items as our within subject experiments – with the exception that participants read aloud or read silently each item. This means that participants in the aloud condition of Experiments 1b and 2b will have produced twice as many items as participants in Experiments 1a and 2a. It could be that producing such a large number of items results in the items becoming relatively less distinctive than their within-subject counterparts.

To address these concerns, we ran three replications of Experiment 2b (i.e., using confidence ratings) via a custom web application: The first “standard” version of our task was a direct replication. The second “short” version of our task was identical to the first except that participants studied half as many items (i.e., 60 study trials rather than 120 study trials). The purpose of this version was to examine the impact that the number of items read aloud or read silently would have on the magnitude of the production effect. The third “filler” version of our task was identical to our “short” version – meaning that participants were presented with only 60 study phase trials – but with the inclusion of an addition 60 filler trials in which the study word was replaced with a variable string of XXXX’s. The purpose of this version was to match the total duration of our between-subject task to our “standard” condition while retaining the same number of study phase trials overall.

However, as mentioned in-text, the three spacing conditions failed to differ from one-another across any of our models. For our analysis of “old” responses (i.e., re-scoring each 1, 2, or 3 response as “New” and each 4, 5, or 6 response as “Old”) all three groups demonstrated a tendency for greater sensitivity in the aloud condition than the silent condition (although this difference was not credible in any individual group) with inconsistent differences in terms of response bias. Notably, the group receiving filler trials demonstrated slightly better performance overall than the other two groups, but this difference was also not credible. The same is generally true for our measures of recollection and familiarity: For recollection, each group demonstrated production effects centered around 0, whereas for familiarity each group tended towards a difference favoring the produced items. These models are depicted in Figure S1. Due to the equivocal between-group differences we opted to collapse these groups in-text to satisfy our other goal for this experiment: To provide a large-scale replication of our between-subject production effect for familiarity, but not recollection, to solidify our findings and provide an additional effect for inclusion in our meta-analysis.

*Analysis of Post-Experimental Strategy Questionnaires*

Finally, to gain further insight into the subjective experience of production, we explored the memory strategies that subjects reported in the post-experimental questionnaires. In this regard, study and test phase strategy responses are summarized in Tables S2 and S3 for Experiments 1-2 and Experiment 3, respectively. For each, the open-ended strategy questions were coded by independent, blinded research assistants naive as to the purpose of this experiment or the condition of any given participant. They were instructed to read the responses pertaining to strategy and to determine which strategies from a provided list were used by each participant at study, keeping in mind that a given participant could employ more than one strategy. This list included Rote Strategies (e.g., *I repeated the word over and over again in my head*), Self-Relational Strategies (e.g., *I related the word to myself or someone I know*), Categorical Strategies (e.g., *I groups the words into categories*), Sensory Strategies (e.g., *I visualized the word or thought of a sound relevant to that word*), Generative Strategies (e.g., *I used the word to generate a story*), Lexical Strategies (e.g., *I focused on how common/unusual the word was or on the form of the word itself*), Miscellaneous Strategies (e.g., *Related the word to a popular song or used my gut to guide my processing*) and No Strategy (e.g., *I did nothing in particular to remember the words*). For those conditions including produced items, coders were then instructed to code how many participants freely volunteered having strategically having retrieved whether they had produced a given study item at test as a means of discriminating between old and new items. For Experiments 1 and 2, they then coded specific questions pertaining to whether participants used access to an existing production trace to guide responses at test and also coded whether participants reported attending more to the green or purple words; in the latter case, this was recoded as whether participants attended more to the aloud or silent words although it is worth reporting that for the between-subject conditions – where color had no meaning – participants appeared to equally endorse paying attention to green words (N = 8 and 10 for Experiments 1b and 2b) and purple words (N = 11 and 6 for Experiments 1b and 2b) with the majority indicating no attentional preference (N = 20 and 30 indicating neither for Experiments 1b and 2b). For Experiment 3, we instead aggregated the Likert scale questions pertaining to (a) how frequently participants reported being able to retrieve having read aloud a given word at study, (b) how frequently participants reported using having read a word aloud as a means of guiding test performance, and, (c) how much attention participants allocated to the study trials overall.

Our interest was in impressionistically evaluating the relative frequency of participants reporting a distinctiveness-based response strategy for within- and between-subject designs, and also to evaluate whether participants subjectively felt that they were paying greater attention to those items they read aloud relative to those they read silently. Whereas few participants volunteered retrieving having produced an item as a means of guiding test performance during the open-ended questions (only between 5% and 13% across our experiments), when specifically asked (*did you try to remember whether you said any of the words as a strategy when tested? If so, why?*) the majority of participants reported doing so regardless of study design (60.00% and 52.00% reported doing so in Experiments 1a and 2a whereas 63.16% and 75.00% reported doing so in Experiments 1b and 2b); similarly, participants in the aloud condition of Experiment 3 reported scores well above the midpoint of the rating scale with respect to being able to recall having spoken a word aloud (*M* = 4.36, *SE* = 0.09) or making use of this information in their recognition responses (*M* = 4.07, *SE* = 0.12); in fact, 70% of participants responded above the midpoint of the scale and 46% of participants selected the most extreme responses (i.e., 5 or 6). Further, practically every participant in Experiments 1a and 2a reported paying greater attention to those items they had been instructed to read aloud relative to those they had been instructed to read silently (92.00% and 80.00% for Experiments 1a and 2a, respectively). In short, participants report greater engagement with the aloud trials relative to the silent trials – a finding supported by one recent study that found participants had a greater propensity to mind-wander while reading passages silently than when reading them aloud (Varao Sousa, Carriere, & Smilek, 2013). In Experiment 3 minimal difference was observed between self-reported attention to the study phase trials (on a 6-point scale) between the silent or aloud condition, and if anything the pattern was in the opposite direction, albeit not credibly so (Aloud - Silent = -0.37, HDI_95%_ = -0.79, 0.05). This slight tendency in favour of silent items might represent participants in the aloud group misinterpreting the question as how much attention was allocated to the word onscreen as opposed to the act of reading it. Of course, it is perhaps difficult to interpret differences in self-reported attention between-groups on the basis that different groups might interpret attention differently due to task differences. Further research is required, perhaps using online measurements integrated into the study phase trials more analogous to those employed by Varao Sousa et al. (2013).

**Supplementary Frequentist Models**

While we maintain that the statistical approach reported in-text is preferable in many ways to a Frequentist analysis of variance (ANOVA), we recognize that not everyone is familiar with this approach. This section therefore presents a re-analysis of much of the data within each experiment using a series of between-subjects, repeated-measures or mixed-effects ANOVAs. We finish by conducting random-effects meta-analyses analogous to our Bayesian meta-analytic models. Except where specifically noted, our conclusions are the same as reported in-text. All ANOVAs were calculated using the *ez* package within *R* (Lawrence, 2013) and our meta-analyses were conducted using the *rma* function from the *metafor* package (Viechtbauer, 2010).

*Experiment 1a*

*Old Responses.* We first collapsed the remember and know responses into binary hits and false alarms for analysis as a function of Item Type (foil, silent, aloud) using a repeated-measures ANOVA; these data are displayed in Table S4. The effect of Item Type was significant, *F*(2,48) = 111.87, *MSe* = 0.01, *p* < .001, η_G_ = .620. Whereas participants committed an average of .21 (*SE* = .02) false alarms, Fisher’s Least Significant Differences (*LSD* = .06; Williams & Abdi, 2010) revealed performance to be significantly higher for both the silent (*M* = .52, *SE* = .03) and aloud conditions (*M* = .65, *SE* = .03), which themselves also differed. Framing our results in terms of the metrics of signal detection theory produces a similar pattern: Participants exhibited greater sensitivity (*d’*) to items read aloud (*M* = 1.34, *SE* = 0.11) than to items read silently (*M* = 0.96, *SE* = 0.10; *t*(24) = 5.55, *p* < .001). For this comparison as well as all other comparisons from Experiments 1a and 1b, similar analyses using untransformed difference scores (i.e., hits – false alarms) produced the same pattern as those using *d’*.

*Remember Responses.* An analogous ANOVA was then used to analyze the proportion of remember responses. This analysis was also significant, *F*(2,48) = 74.24, *MSe* = 0.01, *p* < .001, η_G_ = .545. Whereas participants reported an average of .03 (*SE* = .01) false recollections in response to the foil items, Fisher’s Least Significant Differences (*LSD* = .06) revealed recollection to be significantly higher for both the silent (*M* = .22, *SE* = .02) and aloud conditions (*M* = .37, *SE* = .04), which themselves also differed. Likewise, participants exhibited greater sensitivity (*d’*) to items read aloud (*M* = 1.63, *SE* = 0.10) than to items read silently (*M* = 1.15, *SE* = 0.09; *t*(24) = 6.21, *p* < .001).

*Know Responses.* As described in-text, we were concerned regarding the dependency known to exist between know and remember responses resulting from the fact that as the relative incidence of the latter response increases, there is less opportunity for the former responses to be made. Therefore, we adopted the independent remember/know method (e.g., Jacoby, Yonelinas, & Jennings, 1997; Mangels, Picton, &Craik, 2001;Ochsner, 2000; Yonelinas & Jacoby, 1995). Put simply, the proportion of “Know” responses for each subject were calculated using the formula: *p*(know) / (1 - *p*(remember). An analogous ANOVA was then used to analyze the resulting metric. This analysis was also significant, *F*(2,48) = 44.52, *MSe* = 0.01, *p* < .001, η_G_ = .333. Whereas participants reported an average of .18 (*SE* = .02) familiarity based false alarms, Fisher’s Least Significant Differences (LSD=.06) revealed performance to be significantly higher for both the silent (*M* = .38, *SE* = .03) and aloud (*M* = .44, *SE* = .04) conditions, which also differed. Likewise, participants exhibited greater sensitivity (*d’*) to items read aloud (*M* = 0.86, *SE* = 0.11) than to items read silently (*M* = 0.71, *SE* = 0.09; *t*(24) = 2.34, *p* = .028).

*Experiment 1b*

*Old Responses.* Due to the presence of separable false alarm rates for our conditions of interest, we forego analysis of the raw scores instead calculating *d’* and submitting it to a between-subject ANOVA with Production (silent, aloud) as our factor of interest; these data as well as the raw means are available in Table S4. Although *d’* was greater for the aloud condition relative to the silent condition, this difference was only marginally significant, *F*(1,35) = 3.06, *MSe* = 0.32, *p* = .089, η_G_ = .080.

*Remember Responses.* A similar analysis was undertaken for the remember responses: Specifically, *d’* scores were calculated as before, although in this case five participants failed to make any recollective false alarms. In those cases, a proportion of .01 was used instead to permit calculation of this metric; again, the raw means as well as the calculated *d’* scores are provided in Table S4. In this case, performance was similar in the silent and aloud groups, which did not significantly differ, *F*(1,35) = 0.01, *MSe* = 0.44, *p* = .913, η_G_ < .001.

*Know Responses.* Similar analyses were conducted for the know responses, although in this case proportions were calculated for each condition after excluding trials for which a remember response had been made as described above. These proportions were then used to calculate the *d’* scores that were analyzed and reported in Table S4. Unlike the remember responses, sensitivity was greater for know responses within the aloud group compared to the silent group, *F*(1,35) = 5.23, *MSe* = 0.28, *p* = .028, η_G_ = .130.

*Experiment 2a*

*Old Responses.* Although not of primary interest, we converted our confidence ratings into binary responses by re-scoring each 1, 2, or 3 response as new and each 4, 5, or 6 response as old. These scores were then used to calculate *d’* for analysis. Those scores are reported in Table S5 along with the raw proportion of new and old responses. As depicted there, participants were better able to discriminate those items they read aloud compared to those they read silently, *t*(24) = 5.46, *p* < .001.

*Recollection.* We next analyzed the estimated proportion of recollection trials as a function of production; the estimated values are provided in Table S5. In short, reading a word aloud resulted in a significantly greater proportion of recollective experiences than reading a word silently, *F*(24) = 19.81, *MSe* = 0.01, *p* < .001, η_G_ = .166.

*Familiarity.* The familiarity estimates reported in Table S5 were likewise compared across production condition. As with the recollection estimates, reading a word aloud resulted in significantly greater familiarity than reading a word silently, *F*(24) = 6.60, *MSe* = 0.10, *p* = .017, η_G_ = .064.

*Experiment 2b*

*Old Responses.* As for Experiment 2a, we first converted our confidence ratings into a binary response to permit the calculation of *d’* scores for analysis. Raw means as well as *d’* scores are provided in Table S5. In this instance the supplementary analyses deviate slightly from those reported in-text: Whereas the models reported in-text found a credible difference with regards to sensitivity in the aloud and silent groups – with greater sensitivity for the aloud compared to the silent group – this difference was only marginal in the current analyses, *F*(1,42) = 3.83, *MSe* = 0.17, *p* = .057, η_G_ = .083.

*Recollection.* We next analyzed the estimated proportion of recollection trials as a function of production; the estimated values are provided in Table S5. In short, unlike in Experiment 2a the difference between the aloud and silent groups failed to reach significance, *F*(1,42) = 2.13, *MSe* = 0.02, *p* = .152, η_G_ = .048.

*Familiarity.* Finally*,* we analyzed the familiarity estimates in a similar manner. As depicted in Table S5, despite the use of a between-subject design, reading a word aloud resulted in significant greater familiarity than reading a word silently, *F*(42) = 6.42, *MSe* = 0.11, *p* = .015, η_G_ = .133.

*Experiment 3*

Although Experiment 3 manipulated the number of studied items and spacing of the study phase trials such that they sometimes included fewer items or filler trials, this variable failed to interact with our production manipulation (all *p*’s > .50); we therefore collapsed across this variable within each analysis. The raw scores are nonetheless provided in Table S6.

*Old Responses.* As for Experiment 2b, we first converted our confidence ratings into a binary response to permit the calculation of *d’* scores for analysis. As in the preceding experiment, participants having studied the items aloud were significantly better able to discriminate between new and old items at test, *F*(1,267) = 4.77, *MSe* = 0.71, *p* = .030, η_G_ = .018.

*Recollection.* As in Experiment 2b the difference in recollection between the aloud and silent groups failed to reach significance, *F*(1,267) < 0.01, *MSe* = 0.05, *p* = .945, η_G_ < .001.

*Familiarity.* Unlike recollection, reading a word aloud resulted in significantly greater familiarity than reading a word silently, *F*(1,267) = 10.23, *MSe* = 0.50, *p* = .001, η_G_ = .037.

*ROC Curve Details*

ROC curves involve plotting hit and false alarm rates across varying levels of confidence. So for example, from a 6-point confidence scale one could presume that confidence responses from 2-6 represent extremely lax “old” responses, and thus hit and false alarms could be calculated at this lax criterion. One could then re-consider the data, presuming that confidence responses from 3-6 represent a stricter “old” response, and thus, hit and false alarms could be re-calculated. Following this procedure forward until only confidence responses of 6 are considered “old” responses, the result is a series of hit and false alarm rates calculated from criteria that varied in strictness.^^[[1]](#footnote-1)^^ Plotting ROC curves involves plotting each hit/false alarm pair as a single point on a figure wherein hits are plotted along the y-axis and false alarms along the x-axis. ROC curves for Experiment 2a are shown in Figure S2, plotted separately for aloud and silent items. Similarly, curves for Experiment 3 are shown in Figure S3. For visualization purposes, a curve of best fit is plotted for each aloud and silent items. The curve was derived by fitting the mean confidence data using the DPSD model (see Yonelinas, 1994, 1997). Analyzing ROC curves using the DPSD model, separate estimates of recollection and familiarity can be derived. Hence, ROC curves are presented mainly for visualization purposes only and were not analyzed in great detail. The most relevant analyses are those presented in the main text, which investigated the recollection and familiarity parameter estimates derived from the DPSD model’s fit of subject’s ROC data. It is worthwhile to note however that in the short condition of Experiment 3 the aloud and silent groups showed little difference in their ROC curves. Considering that the short condition represents a typical production effect design, just with fewer study trials than our base condition (but still with a reasonable number of study trials in general, looking at other production effect experiments), at the present time we have no compelling explanation as to why the aloud and silent ROC curves were so similar.

*Meta-analyses*

As our final supplementary analyses, we conducted random-effects meta-analyses analogous to the Bayesian models reported in-text using the *rma* function from the *metafor* package (Viechtbauer, 2010). Effect sizes were calculated using the procedure reported in-text. The findings depicted in Figure S4 agree with those reported in Figure 5: Whereas a clear production effect emerges for the familiarity analysis, with minimal evidence of design effects (Within - Between = 0.06, CI_95%_ = -0.47, 0.34), it is clear that production has a greater influence on recollection when manipulated between-subjects (Within - Between = 0.73, CI_95%_ = 0.32, 1.13) with little effect of production on recollection in between-subject designs. The only qualitative difference between Figure S4 and Figure 5 insofar as the statistical conclusions are concerned is the fact that the effect of production on recollection aggregated across study design (i.e., the polygon at the bottom of the top panel within each figure) just fails to exclude 0 for the Bayesian models but is significant for the Frequentist models. Nonetheless, the aggregate effect is not meaningful given the strong design effects for the recollection model.

**References**

Barr, D. J., Levy, R., Scheepers, C., & Tily, H. J. (2013). Random effects structure for confirmatory hypothesis testing: Keep it maximal. *Journal of Memory and Language*, *68*(3), 255–278. doi:10.1016/j.jml.2012.11.001

Dixon, P. (2008). Models of accuracy in repeated-measures designs. *Journal of Memory and Language*, *59*(4), 447–456. doi:10.1016/j.jml.2007.11.004

Gelman, A., & Hill, J. (2007). *Data analysis using regression and multilevel/hierarchical models.* Cambridge University Press: Cambridge, UK.

Hoffman, M. D., & Gelman, A. (2014). The no-u-turn sampler: Adaptively setting path lengths in Hamiltonian Monte Carlo. *Journal of Machine Learning Research, 15*, 1351–1381.

Kruschke, J. (2010). *Doing Bayesian data analysis: A tutorial with R and BUGS.* Elsevier: Amsterdam.

Lawrence, M. A. (2013). *ez: Easy analysis and visualization of factorial experiments.* R package version 4.2-2. http://CRAN.R-project.org/package=ez

Sorensen, T. & Vasishth, S. (Submitted). *Fitting linear mixed models using JAGS and Stan: A tutorial.*

Stan Development Team (2013). Stan: A C++ Library for Probability and Sampling, Version 2.2.0. URL: <http://mc-stan.org/>.

Williams, L., & Abdi, H. (2010). Fisher's least significant difference test. In N. Salkind (Ed.), *Encyclopedia of research design* (pp. 492e495). Thousand Oaks, CA: Sage Publications, Inc.

Table S1. *The word list used in each of the reported experiments.*

| ability | bosom | city | dirt | garden | jail | metal | priest | shame | time |
| --- | --- | --- | --- | --- | --- | --- | --- | --- | --- |
| affection | boss | claw | disaster | gentleman | joke | mind | prison | sickness | toast |
| air | boulder | clock | disease | glory | journal | misery | procession | situation | tobacco |
| ambassador | bowl | code | disposition | goddess | judge | moisture | product | skin | tomb |
| amount | brain | colony | door | grandmother | jury | moment | pupil | sky | tower |
| animal | butter | committee | drama | grass | justice | monarch | quality | slave | tribute |
| ankle | butterfly | comparison | duty | grief | king | money | queen | slipper | trouble |
| anxiety | cabin | competition | earth | hammer | lake | moral | quest | soil | valley |
| appearance | camp | comrade | effort | harness | lark | moss | railroad | sovereign | vanity |
| arm | capacity | confidence | elephant | hatred | law | murder | reflection | speech | vapor |
| army | captive | conquest | excuse | headquarters | leader | nail | revolt | spray | vegetable |
| array | car | contents | facility | hint | lecture | necessity | river | star | vessel |
| artist | cash | convention | fire | honor | length | nephew | robber | steam | victim |
| assault | cat | corner | fisherman | hope | library | ocean | rock | stone | virtue |
| atmosphere | cellar | costume | flesh | horse | link | oven | rod | storm | vision |
| attendant | chair | cotton | foam | idea | love | oxygen | salute | string | warmth |
| attitude | chance | creature | folly | impulse | majority | party | sauce | substitute | weapon |
| attribute | charm | custom | forest | inhabitant | maker | person | scarlet | sugar | welfare |
| baby | charter | death | fork | instance | marriage | physician | science | sulphur | wheat |
| banker | child | decree | form | institute | master | picture | sea | sunset | wife |
| baron | chin | deed | fowl | intellect | material | plain | sensation | tablespoon | window |
| beaver | Christmas | democracy | fox | interest | meeting | poetry | series | thief | wine |

Table S2. *Memory* *Strategies Reported During Experiments 1 and 2 and Responses to Questions Pertaining to the Heuristic Use of Production at Test and Attentional Allocation at Study. Please Note that A Given Participant Could Employ Multiple Strategies and Therefore These Values Need Not Summate to 100%. Cells Marked with a Dash were Not Applicable Given the Group in Question.*

|  | **E1: Mixed** | **E1: Silent** | **E1: Aloud** | **E2: Mixed** | **E2: Silent** | **E2: Aloud** |
| --- | --- | --- | --- | --- | --- | --- |
| **Strategies** |  |  |  |  |  |  |
| **Rote** | 32.00% | 40.00% | 15.79% | 28.00% | 50.00% | 25.00% |
| **Self-Referential** | 48.00% | 40.00% | 63.16% | 36.00% | 31.82% | 29.27% |
| **Categorical** | 20.00% | 40.00% | 15.79% | 20.00% | 40.91% | 33.33% |
| **Generative** | 16.00% | 20.00% | 10.53% | 16.00% | 4.55% | 20.83% |
| **Sensory** | 8.00% | 20.00% | 26.32% | 24.00% | 27.27% | 20.83% |
| **Lexical** | 4.00% | 20.00% | 10.53% | 8.00% | 18.18% | 8.33% |
| **Miscellaneous** | 8.00% | 25.00% | 10.52% | 20.00% | 9.09% | 4.17% |
| **No Strategy** | 8.00% | 0.00% | 5.26% | 8.00% | 4.55% | 4.17% |
|  |  |  |  |  |  |  |
| **Reported Retrieving Production at Test (Open Question)** | 8.00% | – | 5.26% | 8.00% | – | 12.50% |
| **Reported Retrieving Production at Test (Specific Question)** | 60.00% | – | 63.16% | 52.00% | – | 75.00% |
|  |  |  |  |  |  |  |
| **Reported Paying More Attention to the Aloud Items** | 92.00% | – | – | 80.00% | – | – |

Table S3. *Memory* *Strategies Reported During Experiment 3 and Responses to Questions Pertaining to the Heuristic Use of Production at Test and Attentional Allocation at Study. Please Note that A Given Participant Could Employ Multiple Strategies and Therefore These Values Need Not Summate to 100%.*

|  | **Aloud** | **Silent** | **Aloud-Short** | **Silent-Short** | **Aloud-Filler** | **Silent-Filler** |
| --- | --- | --- | --- | --- | --- | --- |
| **Strategies** |  |  |  |  |  |  |
| **Rote** | 37.78% | 34.69% | 42.22% | 53.33% | 46.51% | 41.03% |
| **Self-Referential** | 24.44% | 16.32% | 11.11% | 22.22% | 23.08% | 25.58% |
| **Categorical** | 13.33% | 20.41% | 17.78% | 6.67% | 20.51% | 13.95% |
| **Generative** | 20.00% | 32.65% | 31.11% | 28.89% | 35.90% | 18.60% |
| **Sensory** | 8.89% | 10.20% | 22.22% | 13.33% | 17.95% | 16.28% |
| **Lexical** | 11.11% | 8.16% | 13.33% | 8.89% | 12.82% | 6.97% |
| **Miscellaneous** | 42.22% | 32.65% | 15.56% | 17.78% | 33.33% | 20.90% |
| **No Strategy** | 4.44% | 0.00% | 8.89% | 2.22% | 0.00% | 0.00% |
|  |  |  |  |  |  |  |
| **Reported Retrieving Production at Test (Open Question)** | 13.33% | – | 6.66% | – | 7.69% | – |
| **Reported Retrieving Production at Test (from 1-6)** | 4.36 (0.13) | – | 4.14 (0.16) | – | 4.59 (0.17) | – |
| **Strategic Use of Production Information at Test (from 1-6)** | 4.18 (0.15) | – | 3.95 (0.23) | – | 4.05 (0.25) | – |
|  |  |  |  |  |  |  |
| **Overall Attention to Study Phase Trials (from 1-6)** | 3.80 (0.27) | 4.14 (0.21) | 4.01 (0.30) | 4.46 (0.22) | 4.94 (0.27) | 5.37 (0.16) |

Table S4. *Mean Proportion (and Standard Error) of Old, Remember and Know Responses and Sensitivity (d’) Estimates for Experiments 1a-b.*

|  | **“Old”** | **“Remember”** | **“Know”** | ***d’_Old_*** | ***d’_Remember_*** | ***d’_Know_*** |
| --- | --- | --- | --- | --- | --- | --- |
| **Experiment 1a** |  |  |  |  |  |  |
| **Foil** | .21 (.02) | .03 (.01) | .18 (.02) | – | – | – |
| **Silent** | .52 (.03) | .22 (.03) | .39 (.03) | 0.96 (0.10) | 1.15 (0.10) | 0.71 (0.09) |
| **Aloud** | .65 (.03) | .37 (.04) | .44 (.04) | 1.34 (0.11) | 1.63 (0.09) | 0.86 (0.11) |
|  |  |  |  |  |  |  |
| **Experiment 1b** |  |  |  |  |  |  |
| **Silent-Foil** | .21 (.03) | .04 (.01) | .18 (.03) | – | – | – |
| **Silent-Target** | .57 (.04) | .29 (.04) | .39 (.04) | 1.08 (0.10) | 1.34 (0.12) | 0.73 (0.10) |
| **Aloud-Foil** | .15 (.02) | .03 (.01) | .13 (.02) | – | – | – |
| **Aloud-Target** | .60 (.03) | .27 (.03) | .46 (.03) | 1.40 (0.09) | 1.36 (0.10) | 1.13 (0.08) |

Table S5. *Raw Proportion (and Standard Error) of Old Responses, Mean Confidence Ratings and Estimates of Recollection and Familiarity for Experiments 2a-b.*

|  | **“Old”** | **Confidence** | ***d’_Old_*** | **Recollection** | **Familiarity** |
| --- | --- | --- | --- | --- | --- |
| **Experiment 2a** |  |  |  |  |  |
| **Foil** | .23 (.02) | 2.56 (0.08) | – | – | – |
| **Silent** | .55 (.03) | 3.78 (0.11) | 0.93 (0.10) | .10 (.03) | 0.86 (0.09) |
| **Aloud** | .72 (.02) | 4.44 (0.09) | 1.42 (0.10) | .18 (.04) | 1.09 (0.10) |
|  |  |  |  |  |  |
| **Experiment 2b** |  |  |  |  |  |
| **Silent-Foil** | .26 (.02) | 2.62 (0.09) | – | – | – |
| **Silent-Target** | .67 (.02) | 4.35 (0.08) | 1.14 (0.06) | .33 (.03) | 0.68 (0.07) |
| **Aloud-Foil** | .17 (.02) | 2.34 (0.09) | – | – | – |
| **Aloud-Target** | .64 (.02) | 4.16 (0.11) | 1.38 (0.06) | .27 (.03) | 0.93 (0.07) |

Table S6. *Raw Proportion (and Standard Error) of Old Responses, Mean Confidence Ratings and Estimates of Recollection and Familiarity for Experiment 3.*

|  | **“Old”** | **Confidence** | ***d’_Old_*** | **Recollection** | **Familiarity** |
| --- | --- | --- | --- | --- | --- |
| **Normal** |  |  |  |  |  |
| **Silent-Foil** | .24 (.02) | 2.47 (0.09) | – | – | – |
| **Silent-Target** | .67 (.02) | 4.35 (0.09) | 1.34 (0.13) | .35 (.03) | 0.73 (0.07) |
| **Aloud-Foil** | .20 (.02) | 2.31 (0.10) | – | – | – |
| **Aloud-Target** | .70 (.02) | 4.42 (0.09) | 1.54 (0.11) | .33 (.03) | 1.00 (0.10) |
|  |  |  |  |  |  |
| **Short** |  |  |  |  |  |
| **Silent-Foil** | .15 (.02) | 2.08 (0.07) | – | – | – |
| **Silent-Target** | .68 (.02) | 4.40 (0.10) | 1.65 (0.11) | .38 (.03) | 0.99 (0.09) |
| **Aloud-Foil** | .17 (.03) | 2.15 (0.11) | – | – | – |
| **Aloud-Target** | .71 (.02) | 4.46 (0.09) | 1.79 (0.12) | .37 (.03) | 1.17 (0.11) |
|  |  |  |  |  |  |
| **Filler** |  |  |  |  |  |
| **Silent-Foil** | .20 (.02) | 2.27 (0.10) | – | – | – |
| **Silent-Target** | .73 (.02) | 4.63 (0.11) | 1.70 (0.13) | .38 (.04) | 1.22 (0.10) |
| **Aloud-Foil** | .15 (.02) | 2.06 (0.09) | – | – | – |
| **Aloud-Target** | .76 (.02) | 4.73 (0.11) | 2.05 (0.15) | .41 (.04) | 1.51 (0.15) |
|  |  |  |  |  |  |
| **Combined** |  |  |  |  |  |
| **Silent-Foil** | .20 (.01) | 2.28 (0.05) | – | – | – |
| **Silent-Target** | .69 (.01) | 4.46 (0.06) | 1.56 (0.07) | .37 (.02) | 0.94 (0.05) |
| **Aloud-Foil** | .17 (.01) | 2.18 (0.06) | – | – | – |
| **Aloud-Target** | .72 (.01) | 4.53 (0.06) | 1.78 (0.07) | .37 (.02) | 1.21 (0.07) |

*Figure S1.* The left column depicts the predicted proportion of “Old” responses and estimates of recollection and familiarity for Experiment 3 as a function of Group (Normal, Filler, Short), Production (Silent, Aloud) and/or Item Type (Foil, Target). The right column depicts the pair-wise contrasts calculated between each of these conditions; thick lines represent the 50% HDI and thin lines represent the 95% HDI. Polygons depict the posterior distribution for each contrast. Please note that each row is on a different scale.

*Figure S2.* The receiver operating characteristics (ROCs) for aloud and silent words in Experiments 2a (within-subjects) and 2b (between-subjects). The displayed curves were fit using the dual-process signal detection model.

| 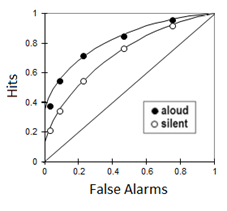  Experiment 2a: within-subjects | 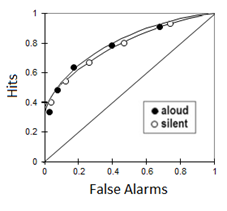  Experiment 2b: between-subjects |
| --- | --- |

*Figure S3*. The receiver operating characteristics (ROCs) for aloud and silent words in Experiment 3. The displayed curves were fit using the dual-process signal detection model. Panel A plots all data from Experiment 3, aggregated over group. Panels B, C, and D plot ROC curves for data from the Aloud/Silent, Aloud/Silent-Filler, and Aloud/Silent-Short groups respectively.

| 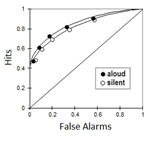All Conditions | 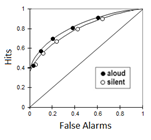Aloud/Silent | 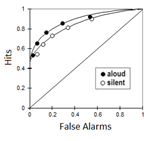Aloud/Silent-Filler | 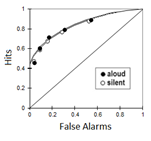Aloud/Silent-Short |
| --- | --- | --- | --- |

*Figure S4.* Forest plots aggregating recollection (top) and familiarity (bottom) estimates from the current experiments as well as those reported by Ozubko et al. (2012) and an unpublished pilot study conducted by Roddick et al. (2014). Effect sizes were calculated as Hedges’ *g* (Hedges, 1981; Morris & DeShon, 2002) and submitted to two separate models: Polygons at the bottom of each plot represent the aggregate effect (and 95% CI) estimated from a model intermixing the within- and between-subject experiments; the remaining polygons represent the aggregate effects (and 95% CIs) estimated from a model incorporating study design (within, between) as a moderator.

1. Note that one never considers the case where confidence responses 1-6 are “old” responses because this would not be a meaningful consideration. Specifically, as there would be no “new” responses if all confidence responses were considered “old”, both hits and false alarms would always equal 1 for any dataset. [↑](#footnote-ref-1)
